# Supplementary material for: Suffering out of sight but not out of mind – interpreting experiences of sick leave due to chronic pain in a community setting: a qualitative study
Source: BMJ Open. 2023 Apr 11;13(4):e066617. doi: 10.1136/bmjopen-2022-066617 (PMC10106073; doi:10.1136/bmjopen-2022-066617)
Supplement: Supplementary data [file bmjopen-2022-066617supp001.pdf]

GÖTEBORGS UNIVERSITET

SAHLGRENSKA ACADEMY

➢ Introduction

Presentation of the researchers, the project and its aim. Information about confidentiality, consent and withdrawal. Opportunity for the participants to ask questions.

➢ Opening questions

- I understand you have been living in pain for a long time. Would you tell me about what this time has been like for you?
- In what way has being on sick leave and not being able to work affected your life?
- What would you say has been the biggest challenge for you as the person you think you are or want to be? Has being on sick leave changed the way you look at yourself?
- Are there things that could have prevented you from being on sick leave?

➢ Work and support

- Having pain for a long time often affects the ability to work. In what way would you say that your pain has influenced your work situation?
- Everyone is different; some people want support to cope with their situation, and others feel they have the support they need to manage. How is it for you? What support did you need to deal with your situation?
- Do you feel that you have been given the opportunity for such support or adjustment at your workplace? If so, what has that support meant to you?

➢ Self-efficacy and confidence in one's ability

- It is common to lose confidence in your abilities when you have been on sick leave for a long time. What do you think about that? Do you feel you can influence your situation yourself?
- Being on sick leave and living with pain can also affect how you feel about your body. Do you feel you can trust your body to do what you want to do? What are your thoughts about that?
- What do you think about the situation you are in today? How does your pain affect the goals and desires that you have in life?

➢ Activity and participation

- Living with pain for a long time can often affect different aspects of everyday life. How do you feel it affects your leisure activities or social contacts?
- How has your pain affected your ability to sleep, rest or recover?

➢ Conclusion

- Do you have any other thoughts or reflections about your situation that we have not discussed today and that you would like to add?
