# Supplementary material for: Suffering out of sight but not out of mind – interpreting experiences of sick leave due to chronic pain in a community setting: a qualitative study
Source: BMJ Open. 2023 Apr 11;13(4):e066617. doi: 10.1136/bmjopen-2022-066617 (PMC10106073; doi:10.1136/bmjopen-2022-066617)
Supplement: Author's manuscript [file bmjopen-2022-066617.draft_revisions.pdf]

BMJ Open

BMJ Open is committed to open peer review. As part of this commitment we make the peer review history of every article we publish publicly available.

When an article is published we post the peer reviewers' comments and the authors' responses online. We also post the versions of the paper that were used during peer review. These are the versions that the peer review comments apply to.

The versions of the paper that follow are the versions that were submitted during the peer review process. They are not the versions of record or the final published versions. They should not be cited or distributed as the published version of this manuscript.

BMJ Open is an open access journal and the full, final, typeset and author-corrected version of record of the manuscript is available on our site with no access controls, subscription charges or pay-per-view fees (http://bmjopen.bmj.com).

If you have any questions on BMJ Open's open peer review process please email info.bmjopen@bmj.com

BMJ Open

Suffering out of sight but not out of mind – a qualitative study of the experiences of being on sick leave due to chronic pain

Journal:	*BMJ Open*
Manuscript ID	bmjopen-2022-066617
Article Type:	Original research
Date Submitted by the Author:	13-Jul-2022
Complete List of Authors:	Lundin, Åse; University of Gothenburg Institute of Health and Care Sciences, Sahlgrenska Academy, University of Gothenburg; University of Gothenburg Centre for Person-Centred Care, Sahlgrenska Academy, University of Gothenburg<br>Ekman, Inger; University of Gothenburg Institute of Health and Care Sciences, Sahlgrenska Academy, University of Gothenburg; University of Gothenburg Centre for Person-Centred Care, Sahlgrenska Academy, University of Gothenburg<br>Wallström, Sara; University of Gothenburg Institute of Health and Care Sciences, Sahlgrenska Academy, University of Gothenburg; University of Gothenburg Centre for Person-Centred Care, Sahlgrenska Academy, University of Gothenburg<br>Andréll, Paulin; Sahlgrenska University Hospital Ostra Hospital, Department of Anaesthesiology and Intensive Care Medicine/Pain Centre; University of Gothenburg Institute of Clinical Sciences, Department of Anaesthesiology and Intensive Care Medicine, Sahlgrenska Academy<br>Lundberg, Mari; Sophiahemmet University, Department of Health Promoting Science; University of Gothenburg Centre for Person-Centred Care, Sahlgrenska Academy, University of Gothenburg
Keywords:	QUALITATIVE RESEARCH, PAIN MANAGEMENT, PUBLIC HEALTH

SCHOLARONE™
Manuscripts

I, the Submitting Author has the right to grant and does grant on behalf of all authors of the Work (as defined in the below author licence), an exclusive licence and/or a non-exclusive licence for contributions from authors who are: i) UK Crown employees; ii) where BMJ has agreed a CC-BY licence shall apply, and/or iii) in accordance with the terms applicable for US Federal Government officers or employees acting as part of their official duties; on a worldwide, perpetual, irrevocable, royalty-free basis to BMJ Publishing Group Ltd ("BMJ") its licensees and where the relevant Journal is co-owned by BMJ to the co-owners of the Journal, to publish the Work in this journal and any other BMJ products and to exploit all rights, as set out in our licence.

The Submitting Author accepts and understands that any supply made under these terms is made by BMJ to the Submitting Author unless you are acting as an employee on behalf of your employer or a postgraduate student of an affiliated institution which is paying any applicable article publishing charge ("APC") for Open Access articles. Where the Submitting Author wishes to make the Work available on an Open Access basis (and intends to pay the relevant APC), the terms of reuse of such Open Access shall be governed by a Creative Commons licence – details of these licences and which Creative Commons licence will apply to this Work are set out in our licence referred to above.

Other than as permitted in any relevant BMJ Author's Self Archiving Policies, I confirm this Work has not been accepted for publication elsewhere, is not being considered for publication elsewhere and does not duplicate material already published. I confirm all authors consent to publication of this Work and authorise the granting of this licence.

For peer review only

Suffering out of sight but not out of mind

- A qualitative study of experiences of being on sick leave due to chronic pain

Word count, excluding title page, abstract, declarations, references and tables: **4452**

Åse Lundin; corresponding author (Orcid ID 0000-0001-9980-7653)
ase.lundin@gu.se
Arvid Wallgrens backe, hus 1
Box 457, 405 30 Göteborg, Sweden

- Institute of Health and Care Sciences, Sahlgrenska Academy, University of Gothenburg, Gothenburg, Sweden
- University of Gothenburg Centre for Person-Centred Care (GPCC), Sahlgrenska Academy, University of Gothenburg, Gothenburg, Sweden

Inger Ekman, Orcid ID: 0000-0002-5559-5203

- Institute of Health and Care Sciences, Sahlgrenska Academy, University of Gothenburg, Gothenburg, Sweden
- University of Gothenburg Centre for Person-Centred Care (GPCC), Sahlgrenska Academy, University of Gothenburg, Gothenburg, Sweden

Sara Wallström: Orcid ID:0000-0001-7579-4974

- Institute of Health and Care Sciences, Sahlgrenska Academy, University of Gothenburg, Gothenburg, Sweden
- University of Gothenburg Centre for Person-Centred Care (GPCC), Sahlgrenska Academy, University of Gothenburg, Gothenburg, Sweden

Paulin Andréll: Orcid ID 0000-0001-6941-6096

- Region Västra Götaland, Sahlgrenska University Hospital/Östra, Department of Anaesthesiology and Intensive Care Medicine/Pain Centre, Gothenburg, Sweden
- Department of Anaesthesiology and Intensive Care Medicine, Institute of Clinical Sciences at Sahlgrenska Academy, University of Gothenburg, Gothenburg, Sweden

Mari Lundberg: Orcid ID 0000-0001-7123-0362

- Department of Health Promoting Science, Sophiahemmet University, Stockholm, Sweden
- University of Gothenburg Centre for Person-Centred Care (GPCC), Sahlgrenska Academy, University of Gothenburg, Gothenburg, Sweden

For peer review only

Abstract

Objectives: Chronic pain is a complex health problem affecting about one-fifth of the European population. It is a leading cause of years lived with disability worldwide with serious personal, relational, and socio-economic consequences. Chronic pain and sick leave adversely affect health and quality of life. Thus, understanding this phenomenon is essential to reduce suffering, understand the need for support, and promote a rapid return to work and an active lifestyle. This study aimed to describe and interpret the experiences of persons living with chronic pain and sickness absence.

Design: A qualitative study with semi-structured interviews analysed using a phenomenological hermeneutic approach.

Participants: Fourteen participants (12 women) who were on part-time or full-time sick leave from work due to chronic pain were included in the study.

Findings: *Suffering out of sight but not out of mind* was the main theme of the qualitative analysis. This theme means that the participants' constant suffering was invisible to others, causing them to feel they were not being justly treated in society. Feeling overlooked led to a continuous struggle for recognition and the participants' identities and their trust in themselves and their bodies was challenged. However, our study also revealed a nuanced understanding of the experiences of sickness absence due to chronic pain where the participants learned important lessons including coping strategies and re-evaluated priorities.

Conclusions: Sickness absence due to chronic pain threatens a person's physical and psychological integrity. Our findings can provide valuable information to health workers, authorities, policymakers, and workplaces about the importance of partnership, support, and recognition that these persons need to be active and healthy working community members.

For peer review only - http://bmjopen.bmj.com/site/about/guidelines.xhtml

Keywords:

Chronic pain, sick leave, qualitative research, phenomenological hermeneutics, lived experiences

Strength and limitations of this study

- This study gave valuable insights into the phenomenon of living with and being on sick leave due to chronic pain, a less studied area of high public health importance
- Qualitative research is important in enlightening the personal experiences of pain and its consequences, uncovering information often not accessible through other methods
- In this study only two out of 14 participants were men, and this distribution might have affected the findings

Introduction

Chronic pain has reached alarming levels, particularly among older adults, and is one of the leading causes of years lived with disability (1). Pain of moderate to severe intensity affects 19% of the European population (2). The socio-economic burden of chronic pain is substantial, mainly for working-age people, with the bulk of indirect costs related to absence from the workforce (3). In the US the total costs associated with persistent pain in adults range from 560–635 billion USD, exceeding the annual costs estimated for heart disease (309 billion USD), cancer (243 billion USD) and diabetes (188 billion USD) (4). In Sweden, the cost of a diagnosis related to chronic pain was estimated to be 32 billion euros per year (3). Chronic pain has a significant impact on quality of life. Suffering from chronic pain is closely associated with depression (5), increased suicide rates (6), premature mortality and reduced life expectancy (7, 8). Because a key feature of pain is that it is a "*personal experience that is*

For peer review only

For peer review only - http://bmjopen.bmj.com/site/about/guidelines.xhtml

influenced to varying degrees by biological, psychological, and social factors" (9), a person's experience or report of pain is of particular interest and should be taken seriously (10). Qualitative research is growing in the field of pain, exposing aspects of experiencing pain that is not accessible through other approaches (11). Previous studies of people's experiences with chronic pain revealed feelings of restriction, loss and limitation of daily life (12, 13), loneliness and silent suffering (14). These studies also showed that chronic pain deeply affected relationships, family life, and work (13). In addition, being on sick leave negatively impacts a person's life. Research on the experiences of being absent from work due to illness has been done in different populations and for different health conditions, revealing negative experiences, including loss of identity (15) social exclusion and a struggle to adapt to reduced work capacity (16). Being in the sick leave process also led to feelings of uncertainty and insecurity (17), stigma, helplessness, and a loss of independence (18).

Commonly, people with chronic pain do not return to work despite genuine efforts to do so (e.g., interdisciplinary rehabilitation) (19). Qualitative pain research has often focused on experiences of barriers and facilitators of return to work for people with chronic pain (20, 21). However, there are limited data on the personal experiences of being in the sick leave process due to chronic pain. Some studies reflect on immigrant women's experiences of long-term sick leave due to chronic pain. The studies found these women felt excluded and rejected (22), struggling with an invisible physical ailment (23). Other studies have explored the phenomenon on a descriptive level (24). Yet, researchers have been encouraged to go beyond description and towards interpretative engagement while performing qualitative analyses of pain (11). In line with this approach, we sought to understand the essential meaning of sickness absence due to chronic pain. A deepened understanding of the personal experiences of the phenomenon is vital in order to reduce suffering, better understand their needs and facilitate a return to working life.

For peer review only - http://bmjopen.bmj.com/site/about/guidelines.xhtml

This study aimed to describe and interpret the experiences of persons living with chronic pain and sickness absence.

Method

The authors followed the Standards for Reporting Qualitative Research (SRQR) guidelines (25) to ensure the trustworthiness of the findings.

Design

In this study we chose a qualitative interview design to explore people's experiences of living with chronic pain and sick leave. Data in the form of transcripts of semi-structured interviews were analyzed using phenomenological hermeneutics inspired by Ricoeur (26) and developed by Lindseth and Norberg (27). The method has been used in numerous health and care studies (e.g., (28-30). The method aims to understand the meaning of the participants' lived experiences by interpreting their narratives.

Participants and setting

Fourteen participants (12 women and 2 men) who complied with inclusion criteria were included in the study and went on to be interviewed. The participants were recruited through patient organizations and associations that focus on pain or pain-related conditions. A request for participation was sent out through their e-mail, newsletter or Facebook pages and 18 people contacted the first author (ÅL) regarding participation. Three decided not to go through with an interview and one did not show for the booked interview. Participant characteristics are described in Table 1.

Table 1

**Participant characteristics**	**(n = 14)**
Men/Women	2/12

For peer review only - http://bmjopen.bmj.com/site/about/guidelines.xhtml

Mean age, years (range)	49 (23-80)
Current work status	
Full-time sick leave	4
Part-time sick leave	5
Returned to work	4
Retired	1
Highest completed education	
Primary school (9 years)	2
High School	3
Other upper secondary education	4
University degree	5

Data collection

Data were collected through individual semi-structured in-depth interviews lasting from 41-90 minutes. The interviews were conducted in 2021 by ÅL and were audio-recorded and transcribed verbatim. All interviews and transcripts were confidential using numerical coding, and precautions were taken to safeguard the identity of the participants. The collected data and narratives that form the basis for analysis consist of about 220 text pages.

Ten interviews were performed through digital channels; two were in the participants' homes and two on the university's premises. The participants were able to choose the interview location. An interview guide was used (see supplemental material), but the participants were asked to narrate their situation freely. Follow-up questions were used to penetrate the aim or whenever the research questions were not addressed spontaneously.

Analysis and interpretation of data

Phenomenological hermeneutics comprises three interwoven parts: naïve reading, structural analysis and interpretation of the whole (27). During the naïve reading, the text was read repeatedly to grasp its meaning as a whole. In the structural analysis the text related to the aim of the study was divided into meaning units that were then abstracted and formed into subthemes, themes and a main theme. NVivo was used in the structural analysis. The analysis was a continuous process moving between the parts and meaning units of the text and the overall understanding. ÅL, ML and IE met regularly during this process to discuss the collected data and the emergence of subthemes, themes and main theme to highlight different perspectives, add breadth and nuances but also to ensure a congruent reflection and

For peer review only - http://bmjopen.bmj.com/site/about/guidelines.xhtml

understanding of the narratives. All parts of the text were considered, but the parts not relevant to the research question were not included in the analysis. During the final part of the analysis, the text was reread, and the initial naïve reading, the structural analysis and the authors' preunderstandings were combined to interpret the text's overall meaning. We used research related to the area, theoretical literature and discussions among the authors to gain a clearer and deeper understanding of the text.

Research characteristics and reflexivity

ÅL is a physiotherapist with several years of clinical experience in primary health care. The author has frequently encountered patients seeking care for chronic pain who cannot work due to their condition. She has previously done qualitative research but not in areas related to pain. Of the co-authors, two have long experience in research on pain (ML and PA) and three have extensive experience working with qualitative analyses (IE, ML, and SW).

Ethical considerations

Written consent was obtained from each participant. The study was approved by the Swedish Ethical Review Authority DNR 2020-02491 and complied with the principles in the Declaration of Helsinki (31).

Patient and public involvement

Before the study was undertaken, several patient associations were contacted to help recruit participants. These patient organizations and their channels will also be used to disseminate the study results to reach a wider audience beyond the academic community.

Findings

The naïve reading of the text revealed that living with chronic pain and being on sick leave significantly impacted the participants' lives and how they identified themselves. The feelings of exclusion, loss and unfamiliarity permeated the naïve understanding. The participants struggled to find ways to manage a condition experienced as invisible to others and hindered participation in daily activities and work.

The structural analyses partly validated our first impressions. These analyses also helped nuance and highlight other parts of the phenomenon, mainly regarding dependency and

For peer review only - http://bmjopen.bmj.com/site/about/guidelines.xhtml

relations with institutions, understandings of the phenomenon that went unnoticed in the first part of the analysis.

An overview of the main theme, themes and subthemes from the structural analysis is given in Table 2 and further described below.

Table 2
Overview of the main theme, themes and subthemes

Suffering out of sight but not out of mind		
**An identity under threat**	**Caught in a foreign body**	**Navigating uncharted waters**
Becoming dependent<br>Being excluded<br>Feeling flawed	Losing bodily trust<br>Feeling constantly tired	Constantly chasing recognition<br>A journey towards unexpected lessons

An identity under threat

Being on sick leave due to chronic pain affected and threatened the participants' views of themselves and their identity, significantly impacting their relationships with others. This theme was expressed in three subthemes: Becoming dependent, Being excluded and Feeling flawed.

1. Becoming dependent

The participants described no longer recognizing themselves concerning their capabilities. They had previously viewed themselves as independent and able to care and fend for themselves. However, sickness absence due to pain forced them to become increasingly dependent on others. Becoming dependent involved having to rely on help from family and friends, outsource physical household chores and become economically reliant on their partners to pay for their dwelling or provide for the children. Participants experienced

For peer review only - http://bmjopen.bmj.com/site/about/guidelines.xhtml

frustration from relying on authorities' benefits and goodwill regarding their work status and sick leave payments. Decisions were often out of the participants' control, causing distress and a feeling of worthlessness and powerlessness. One participant who was denied access to sick leave benefits said,

In the end, you feel like you are just a burden to your husband [crying]. I have no income to get by myself. I cannot earn a living. And if he cannot service or help me or fix the car, then we have to sell it because I can't do it. # 4

2. Being excluded

The presence of pain and being on sick leave led to being left out of the workforce and losing value as an employee and person. The participants previously felt pride in being hard workers and wanted to return to work and be a part of the social life and the routines they had at the workplace that made them feel meaningful and appreciated. Having to go on sick leave was experienced as being imprisoned or punished and a struggle of being left behind.

Being at home it…I think it is the worst punishment. The days…the days are long when you are healthy so you can imagine when you are sick and restricted and…and being alone in the house and my husband is working and my daughter is at school and then you think a lot of negative thoughts and you have to occupy the brain, I guess. #14

3. Feeling flawed

Because the participants lived with pain and could not work, they felt flawed as persons, parents, and participants in daily life. They struggled with a loss of engagement, feeling

For peer review only - http://bmjopen.bmj.com/site/about/guidelines.xhtml

unhappy, increased anger, short temper towards family members, and other personality changes. They often had to decline participation in social activities or hobbies against their wishes. It affected their relationships with family and friends, creating grief, largely concerning being unable to be there for their friends, spouse, or children as they used to be.

> *But you still feel that it's like you are not sufficiently there. So, you also have a constant feeling of guilt that "no, mommy isn't able to…". #6*

For peer review only

Caught in a foreign body

Suffering from chronic pain meant not recognizing the body as the familiar place it used to be, creating conflicts that made it difficult to cope with daily and working life. This theme was expressed in two subthemes: Losing bodily trust and Feeling constantly tired.

1. Losing bodily trust

Experiencing distressing physical symptoms combined with intensive and unreliable pain fluctuations was very tiresome for the participants. Not knowing how their body would function or react from day to day created a loss of trust and a feeling that their body was not the safe and trusted home it used to be. One participant described it as always wearing a heavy diving suit, interpreted as a conflict between what the participant wanted and what the body could do. The loss of bodily trust was exacerbated by being on sick leave, and it also hindered the participants from feeling capable of returning to work and interacting with others.

> *Then I would make supper and help them into their nightclothes, and then you ought to…some of them who were in wheelchairs would have toilet visits and*

For peer review only - http://bmjopen.bmj.com/site/about/guidelines.xhtml

then do many things. And I drop pots and cups on sinks, and it banged and these older people, 80-90 years old, were terrified! And I got so much pain that I could barely lift this pot back up again, or the cup or… I mean, I would have hot, both hot water and stuff. I'm terrified that I would drop an old…/… /And then I did not want to go back there when I know that I do not, that I cannot hold. When I cannot even grab a cup, how can I manage to hold a whole person? # 4

2. Feeling constantly tired

The participants experienced that fatigue and the inability to sleep were a greater disturbance in their daily lives and their ability to work than the pain itself. The little energy possibly gained through the night was quickly drained the next day when coping with the fatigue associated with the pain. Sleepless nights, the side effects of sleep medication, and not feeling rested were associated with great stress and suffering. Sickness absence and not having to get up for work opened up the possibility to rest a little during the day instead of the night, which was interpreted as a small window of opportunity to find relief.

Navigating uncharted waters

Living in pain and being unable to work was interpreted as navigating through the unfamiliar, a route filled with unpleasantness and uncertainties (but sometimes helpful surprises). This theme was expressed in two subthemes: Constantly chasing recognition and A journey towards unexpected lessons.

1. Constantly chasing recognition

For peer review only - http://bmjopen.bmj.com/site/about/guidelines.xhtml

For peer review only

Throughout the narratives, it became clear that living with pain meant facing mistrust from most parts of society. Pain was experienced as a hidden disease by the participants. An invisible condition made them feel invariably questioned, especially in contact with the authorities, the healthcare system, and their workplace. They thought they were met with accusations of being complainers, being lazy, and making up or exaggerating their symptoms. These accusations were interpreted as a constant and never-ending pursuit to be believed and have their lifeworld recognized. The participants continuously fought to receive a diagnosis that could be shown through objective testing to explain their pain and validate their leave of absence from work.

*It does not matter what I say because it feels like they see my smile and that I am active and walking that "well, but then she is not so ill." And if I had gone back to work "but then you are not so ill," and it starts all over again!/…./Just because it is not visible on a damn test…That you couldn't just, you know you can connect cars to computers, couldn't you get a frigging body scan and *swoosh* where it lights up red, she's in pain there! # 3*

2. A journey towards unexpected lessons

The participants were forced to find different coping strategies and new routines because of the profound fatigue and constant pain. This endeavour was interpreted as a bumpy journey with uncertainties and worries, especially regarding the future and how their life and work will be if their condition deteriorates. The participants' experiences of living with pain were not all undesirable in that some had learned important life lessons. They gained new insights about the importance of not pushing oneself too hard, that rest is imperative, and that it is not beneficial to let work dominate life. They identified abilities and resources within themselves

For peer review only - http://bmjopen.bmj.com/site/about/guidelines.xhtml

that they had not previously seen that helped them cope with their new situation. They also re-evaluated their priorities regarding relationships and family and the importance of appreciating the small things in life.

> *I think I'm better at thinking that the small things are good and pleasant. It may be enough that I go for a walk in the woods and look at the water. You will still be all right, or better. In a way that I didn't …I didn't see the qualities of the little things in life quite the same way. Maybe it was the bigger stuff that was more important. The rest was mostly just everyday things. I do not think that way anymore; instead, I believe that there are small things that are important after all. #8*

Interpretation of the whole

The naïve reading, the structural analysis, and the authors' preunderstandings were combined to interpret the overall meaning of the text material. The interpretation was inspired by Ricoeur's philosophy and ethical intentions of *"aiming at a good life with and for others in just institutions"* (32), emphasizing that ethics is prioritized over morality or norms. Ricoeur's philosophy on just institutions is of particular interest to this study as it is essential that people feel justly treated when in contact with institutions (33) such as the healthcare system, the authorities, or the workplace. With pain being experienced as an invisible and questionable illness, there was an incessant pursuit to have their life situation validated and recognized by others. Because of this lack of recognition, the participants felt they were not receiving the justice they deserved. During their sick leave, the participants' identities were threatened and challenged by feelings of flaws, exclusion, and dependency. They felt they were caught in their bodies, which they experienced as foreign, not entirely their own. The

For peer review only - http://bmjopen.bmj.com/site/about/guidelines.xhtml

themes were interpreted as being connected by being processes that were invisible to others, to institutions, but still brought much suffering to the participants. Although their struggles were hidden from the outside world, they were real to the participants, affecting all aspects of life. Based on this, the meaning of living with and being on sick leave due to chronic pain was interpreted as *Suffering out of sight but not out of mind,* which could be regarded as the main theme. It is a play on the idiom "out of sight, out of mind," meaning that you soon forget people (or things) that are no longer visible or present. For the participants, however, the pain and suffering were permanent and real.

Discussion

The main findings of this study are that the participants experienced their suffering as invisible to others, causing them to feel they were not being justly treated in society and constantly had to struggle for recognition. Moreover, being on sick leave due to chronic pain challenged the participants' identities and their trust in themselves and their bodies. Contrary to previous research, which has mainly highlighted the hardships and adversities of sickness absence (16, 17, 22, 23), our study revealed a nuanced understanding of the experiences of being on sick leave due to chronic pain. Despite their difficulties, and sometimes to their surprise, the participants felt that this situation had taught them new things about themselves, forced them to develop new coping-strategies and given them opportunities to embrace other aspects of life. Research has shown that the presence of supportive experiences (e.g., meeting respectful health care professionals) is important to those on sick leave (18) and that possessing problem-solving coping strategies significantly reduces sickness absence (34). In our study we also interpreted the participants' learning experiences as something that stretched over a broader context and brought added value to them regarding relationships,

For peer review only - http://bmjopen.bmj.com/site/about/guidelines.xhtml

family, and having a positive approach to life. They were insights and understanding that would not have been reached without the participants facing the process of illness and sick leave. Our findings are important because if the participants had learned these lessons (e.g., coping strategies) earlier, sick leave might have been shortened or prevented. The participants in our study explained that they might have continued working if their struggles had been recognized at the workplace or by the healthcare system from the start. We interpret this inadequacy as lacking the institutional support they needed, and looking at it in regard to Ricoeur, not met by what would be considered a just institution.

The experience of being in a foreign body when suffering from poor health has been described in other studies. In illness the body can be perceived as unhomelike and can be seen as a "broken tool" that is no longer in control, resulting in a loss of command over one's own body (35). Suffering from chronic pain could also be interpreted as experiencing the body as an obstruction and being confronted with having a body that feels different than when healthy (36). The participants in our study described how they were affected by several neurophysiological changes due to pain including fatigue, stress and mood changes such as increased anger. According to our clinical experiences this might represent a lack of knowledge of the dialectical relationship between the body and psychological or behavioural changes. Increasing the understanding of the bidirectional relationship between the mind and the body and how this interaction affects the pain experience is an important part in pain rehabilitation. Pain neuroscience education has been shown to increase this knowledge and reduce disability among patients suffering from chronic pain (37). Our study also found that the loss of familiarity with the body when suffering from pain hindered the participants' return to work. The ability to return to work was interpreted as not only a question of the physical strength needed but also trust in their capabilities and feeling safe in their bodies in the context of the workplace. As shown in previous research, the experience of moving while

For peer review only - http://bmjopen.bmj.com/site/about/guidelines.xhtml

in persistent pain implies more than just physical restraints. It challenges and threatens a person's entire lifeworld and existence (38). In the words of Merleau-Ponty, our lived body is our access to or *"a vehicle of being in the world"* (39) and when our body changes, or in this case, where the participants felt trapped in an unknown body due to pain, it alters the way the world is perceived.

Our participants viewed their identities as challenged or under threat while on sick leave due to pain. This feeling is consistent with previous findings on the sick leave process due to other health conditions, expressed as being reduced as a person and not recognizing oneself (15) or experiencing a changed self-perception (18). Our participants described a strong connection between their identity and their work. Losing contact with the workplace meant losing who they were as productive persons. The authenticity of pain conditions as a cause of work absence has been questioned by some employers, suggesting that workers are "lazy" (21). In contrast, our study shows a strong desire to return to work and the appreciation they felt was linked to being part of the workforce.

We interpreted the experiences of constantly chasing recognition while facing distrust and suspicion from others as not being met by just institutions. The meaning of a just institution is that each person when in contact with for example the health care system senses justice on a fundamental level, i.e., is treated well and considered equal partners in the care process (33). A just institution is a crucial part of person-centred care and allows persons to express their needs and abilities and even offer a sense of partnership (33). In our study, however, the participants described feeling subservient and helpless, with their lives dependent on the goodwill of institutional gatekeepers. Previous research on sick leave has observed similar experiences, i.e. instead of a partnership, the rules and regulations make the stakeholders become counterparts, leaving little flexibility for the person (15). But being on sick leave due to chronic pain was also interpreted as different from other health issues, mainly because of

For peer review only - http://bmjopen.bmj.com/site/about/guidelines.xhtml

its invisibility and the lack of objective testing of pain symptoms. A social stigma is attached to chronic pain (40) and in our study one participant wondered whether the workplace or authorities would have looked at her and her sick leave in a different light if she had cancer rather than a chronic pain condition?

Methodological considerations

In the current study only two out of 14 participants were men. This skewed distribution might partly be explained by research showing that women report a higher prevalence of chronic pain (41) but could also be influenced by other factors (e.g., women being overrepresented or more active in the patient organizations used here to recruit participants). The study also explored the 14 participants' narratives, and their experiences might not apply to other people on sick leave due to other chronic pain conditions. However, qualitative research does not argue that the findings are universal and generalizable; rather, it explores possible meanings of lived experiences to understand and improve our practices in health care (27).

Sample size in qualitative studies is often a subject of discussion, where no power calculations can determine the sample size needed (42). In phenomenological research sample sizes of between 5 and 25 persons are commonly suggested (43). But determining sample size and when no further sampling is needed is a matter of judgment and experience in evaluating the quality of already collected data to the aims and methods used (42). During the inclusion process and inspection of the collected interview data, the material was discussed within the research group and deemed to contain rich and varied narratives of the experiences, sufficient to answer the research question using the phenomenological hermeneutic method.

Another potential limitation is related to the COVID-19 pandemic restrictions. Because of the restrictions, most interviews were performed digitally, which could have affected the

For peer review only - http://bmjopen.bmj.com/site/about/guidelines.xhtml

interactions and dynamics during the interviews compared to face-to-face encounters. A recent review showed that digital interviews could adversely affect the interviews, including experiencing technical issues or difficulties reading visual cues or body language. The review also found that richer data could be extracted through a digital approach when investigating sensitive topics (e.g., health issues), most likely because the participants felt more in control of the situation and the setting and thus felt more relaxed (44).

Conclusions, clinical implications, and future research

This study underscores the experiences of persons on sick leave due to chronic pain, where feelings of being invisible and overlooked often dominate. They do not feel that they are met with justice in their encounters with their surroundings, creating existential concerns and threatening their identities, bodies, and roles to others. In contrast to previous research which have mainly high-lighted the adversities of being in the sick leave process, our study revealed a nuanced understanding of the participants' learning experiences of sickness absence due to chronic pain. The current results can be helpful on several levels, they can provide information to health care personnel, authorities, policymakers, and workplaces about the importance of partnership and recognition these persons need to be active and healthy working members of the community. Suggestions for future research include further exploring what experiences or factors might play a role in supporting persons on sick leave due to chronic pain in returning to work.

Declarations

For peer review only - http://bmjopen.bmj.com/site/about/guidelines.xhtml

- **Ethics approval**

  The study was approved by the Swedish Ethical Review Authority DNR 2020-02491 and conformed to the Declaration of Helsinki (31). Written informed consent was obtained from each participant at the start of the study.

- **Funding**

  This work was supported by FORTE grant number 2019-00718 and The University of Gothenburg Centre for Person-Centred Care (GPCC) grant number N/A.

- **Contributors**

  All authors participated in the design of the study. ÅL conducted and transcribed the interviews and performed the analysis with critical input from IE and ML. The manuscript was drafted by ÅL and reviewed and edited by IE, ML, SW and PA. All authors read and approved the final manuscript.

- **Competing interests**

  None declared

- **Data sharing statement**

  The interview data are not publicly available.

- **Patient consent form**

  Not required

- **Acknowledgments**

  We acknowledge the participants for taking the time and effort to share their narratives and experiences. Without them, this study would not have been possible.

References

1. Rice ASC, Smith BH, Blyth FM. Pain and the global burden of disease. Pain. 2016;157(4).

For peer review only - http://bmjopen.bmj.com/site/about/guidelines.xhtml

For peer review only

2. Breivik H, Collett B, Ventafridda V, Cohen R, Gallacher D. Survey of chronic pain in Europe: prevalence, impact on daily life, and treatment. European journal of pain (London, England). 2006;10(4):287-333.
3. Gustavsson A, Bjorkman J, Ljungcrantz C, Rhodin A, Rivano-Fischer M, Sjolund K-F, et al. Socio-economic burden of patients with a diagnosis related to chronic pain – Register data of 840,000 Swedish patients. 2012;16(2):289-99.
4. Gaskin DJ, Richard P. The economic costs of pain in the United States. The journal of pain. 2012;13(8):715-24.
5. Bair MJ, Robinson RL, Katon W, Kroenke K. Depression and Pain Comorbidity: A Literature Review. Archives of Internal Medicine. 2003;163(20):2433-45.
6. Tang NKY, Crane C. Suicidality in chronic pain: a review of the prevalence, risk factors and psychological links. Psychological Medicine. 2006;36(5):575-86.
7. Torrance N, Elliott AM, Lee AJ, Smith BH. Severe chronic pain is associated with increased 10 year mortality. A cohort record linkage study. 2010;14(4):380-6.
8. Smith D, Wilkie R, Uthman O, Jordan JL, McBeth J. Chronic Pain and Mortality: A Systematic Review. PLOS ONE. 2014;9(6):e99048.
9. Raja SN, Carr DB, Cohen M, Finnerup NB, Flor H, Gibson S, et al. The revised International Association for the Study of Pain definition of pain: concepts, challenges, and compromises. 2020;161(9):1976-82.
10. Cohen SP, Vase L, Hooten WM. Chronic pain: an update on burden, best practices, and new advances. The Lancet. 2021;397(10289):2082-97.
11. Osborn M, Rodham K. Insights into Pain: A Review of Qualitative Research. Rev Pain. 2010;4(1):2-7.
12. Carson MG, Mitchell GJ. The experience of living with persistent pain. Journal of advanced nursing. 1998;28(6):1242-8.
13. De Souza L, Oliver Frank A. Patients' experiences of the impact of chronic back pain on family life and work. Disability and rehabilitation. 2011;33(4):310-8.
14. Gillsjö C, Nässén K, Berglund M. Suffering in silence: a qualitative study of older adults' experiences of living with long-term musculoskeletal pain at home. European journal of ageing. 2021;18(1):55-63.
15. Andersson C, Mårtensson L. Women's experiences of being in the sick leave process. Scandinavian journal of occupational therapy. 2021;28(6):488-97.
16. Severinsen A, Midtgaard J, Backhausen MG, Broberg L, Hegaard HK. Pregnant women's experiences with sick leave caused by low back pain. A qualitative study. Work (Reading, Mass). 2019;64(2):271-81.
17. Lindbäck C, Nordgren L. To be on sick-leave due to heart failure: a qualitative perspective. Disability and rehabilitation. 2015;37(19):1732-8.
18. Lännerström L, Wallman T, Holmström IK. Losing independence – the lived experience of being long-term sick-listed. BMC Public Health. 2013;13(1):745.
19. LoMartire R, Björk M, Dahlström Ö, Constan L, Frumento P, Vixner L, et al. The value of interdisciplinary treatment for sickness absence in chronic pain: A nationwide register-based cohort study. 2021;25(10):2190-201.
20. Wainwright E, Wainwright D, Keogh E, Eccleston C. Return to work with chronic pain: employers' and employees' views. Occupational Medicine. 2013;63(7):501-6.

For peer review only - http://bmjopen.bmj.com/site/about/guidelines.xhtml

21. Grant M, Rees S, Underwood M, Froud R. Obstacles to returning to work with chronic pain: in-depth interviews with people who are off work due to chronic pain and employers. BMC musculoskeletal disorders. 2019;20(1):486.
22. Nortvedt L, Lohne V, Kumar BN, Hansen HP. A lonely life--A qualitative study of immigrant women on long-term sick leave in Norway. International journal of nursing studies. 2016;54:54-64.
23. Nortvedt L, Hansen HP, Kumar BN, Lohne V. Caught in suffering bodies: a qualitative study of immigrant women on long-term sick leave in Norway. Journal of clinical nursing. 2015;24(21-22):3266-75.
24. Brataas HV, Evensen AE. Life stories of people on sick leave from work because of mild mental illness, pain and fatigue. Work (Reading, Mass). 2015;53(2):285-91.
25. O'Brien BC, Harris IB, Beckman TJ, Reed DA, Cook DA. Standards for Reporting Qualitative Research: A Synthesis of Recommendations. 2014;89(9):1245-51.
26. Ricoeur P. Interpretation Theory: Discourse and the Surplus of Meaning: Texas Christian University Press; 1976.
27. Lindseth A, Norberg A. A phenomenological hermeneutical method for researching lived experience. Scandinavian Journal of Caring Sciences. 2004;18(2):145-53.
28. Alsén S, Ali L, Ekman I, Fors A. Facing a blind alley - Experiences of stress-related exhaustion: a qualitative study. BMJ open. 2020;10(9):e038230.
29. Wallström S, Ulin K, Omerovic E, Ekman I. Symptoms in patients with takotsubo syndrome: a qualitative interview study. BMJ open. 2016;6(10):e011820.
30. Söderberg S, Lundman B, Norberg A. Struggling for dignity: the meaning of women's experiences of living with fibromyalgia. Qualitative health research. 1999;9(5):575-87.
31. Association WM. World Medical Association Declaration of Helsinki: Ethical Principles for Medical Research Involving Human Subjects. JAMA. 2013;310(20):2191-4.
32. Ricœur P. Oneself as another. New ed. ed. Blamey K, editor: Chigago : University of Chicago Press; 1994.
33. Ekman I. Practising the ethics of person-centred care balancing ethical conviction and moral obligations. Nursing Philosophy. 2022:e12382.
34. van Rhenen W, Schaufeli WB, van Dijk FJ, Blonk RW. Coping and sickness absence. International archives of occupational and environmental health. 2008;81(4):461-72.
35. Svenaeus F. The body uncanny — Further steps towards a phenomenology of illness. Medicine, Health Care and Philosophy. 2000;3(2):125-37.
36. Paulson M, Danielson E, Söderberg S. Struggling for a Tolerable Existence: The Meaning of Men's Lived Experiences of Living with Pain of Fibromyalgia Type. 2002;12(2):238-49.
37. Geneen LJ, Martin DJ, Adams N, Clarke C, Dunbar M, Jones D, et al. Effects of education to facilitate knowledge about chronic pain for adults: a systematic review with meta-analysis. Systematic Reviews. 2015;4(1):132.
38. Lundberg M, Styf J, Bullington J. Experiences of moving with persistent pain--a qualitative study from a patient perspective. Physiotherapy theory and practice. 2007;23(4):199-209.
39. Merleau-Ponty M. Phenomenology of Perception: The Humanities Press; 1962.
40. De Ruddere L, Craig KD. Understanding stigma and chronic pain: a-state-of-the-art review. 2016;157(8):1607-10.

For peer review only - http://bmjopen.bmj.com/site/about/guidelines.xhtml

41. Wijnhoven HA, de Vet HC, Picavet HS. Prevalence of musculoskeletal disorders is systematically higher in women than in men. The Clinical journal of pain. 2006;22(8):717-24.
42. Sandelowski M. Sample size in qualitative research. Res Nurs Health. 1995;18(2):179-83.
43. Polkinghorne DE. Phenomenological research methods. Existential-phenomenological perspectives in psychology: Exploring the breadth of human experience. New York, NY, US: Plenum Press; 1989. p. 41-60.
44. Thunberg S, Arnell L. Pioneering the use of technologies in qualitative research – A research review of the use of digital interviews. International Journal of Social Research Methodology. 2021:1-12.

For peer review only

For peer review only - http://bmjopen.bmj.com/site/about/guidelines.xhtml

GÖTEBORGS UNIVERSITET

SAHLGRENSKA ACADEMY

➢ Introduction

Presentation of the researchers, the project and its aim. Information about confidentiality, consent and withdrawal. Opportunity for the participants to ask questions.

➢ Opening questions

- I understand you have been living in pain for a long time. Would you tell me about what this time has been like for you?
- In what way has being on sick leave and not being able to work affected your life?
- What would you say has been the biggest challenge for you as the person you think you are or want to be? Has being on sick leave changed the way you look at yourself?
- Are there things that could have prevented you from being on sick leave?

➢ Work and support

- Having pain for a long time often affects the ability to work. In what way would you say that your pain has influenced your work situation?
- Everyone is different; some people want support to cope with their situation, and others feel they have the support they need to manage. How is it for you? What support did you need to deal with your situation?
- Do you feel that you have been given the opportunity for such support or adjustment at your workplace? If so, what has that support meant to you?

➢ Self-efficacy and confidence in one's ability

- It is common to lose confidence in your abilities when you have been on sick leave for a long time. What do you think about that? Do you feel you can influence your situation yourself?
- Being on sick leave and living with pain can also affect how you feel about your body. Do you feel you can trust your body to do what you want to do? What are your thoughts about that?
- What do you think about the situation you are in today? How does your pain affect the goals and desires that you have in life?

➢ Activity and participation

- Living with pain for a long time can often affect different aspects of everyday life. How do you feel it affects your leisure activities or social contacts?
- How has your pain affected your ability to sleep, rest or recover?

➢ Conclusion

- Do you have any other thoughts or reflections about your situation that we have not discussed today and that you would like to add?

For peer review only

Reporting checklist for qualitative study.

Based on the SRQR guidelines.

Instructions to authors

Complete this checklist by entering the page numbers from your manuscript where readers will find each of the items listed below.

Your article may not currently address all the items on the checklist. Please modify your text to include the missing information. If you are certain that an item does not apply, please write "n/a" and provide a short explanation.

Upload your completed checklist as an extra file when you submit to a journal.

In your methods section, say that you used the SRQRreporting guidelines, and cite them as:

O'Brien BC, Harris IB, Beckman TJ, Reed DA, Cook DA. Standards for reporting qualitative research: a synthesis of recommendations. Acad Med. 2014;89(9):1245-1251.

		Reporting Item	Page Number
**Title**			
	#1	Concise description of the nature and topic of the study identifying the study as qualitative or indicating the approach (e.g. ethnography, grounded theory) or data collection methods (e.g. interview, focus group) is recommended	1
**Abstract**			
	#2	Summary of the key elements of the study using the abstract format of the intended publication; typically includes background, purpose, methods, results and conclusions	2
**Introduction**			
Problem formulation	#3	Description and signifcance of the problem / phenomenon studied: review of relevant theory and empirical work; problem statement	3-5

For peer review only

Purpose or research question	#4	Purpose of the study and specific objectives or questions	5
**Methods**			
Qualitative approach and research paradigm	#5	Qualitative approach (e.g. ethnography, grounded theory, case study, phenomenolgy, narrative research) and guiding theory if appropriate; identifying the research paradigm (e.g. postpositivist, constructivist / interpretivist) is also recommended; rationale. The rationale should briefly discuss the justification for choosing that theory, approach, method or technique rather than other options available; the assumptions and limitations implicit in those choices and how those choices influence study conclusions and transferability. As appropriate the rationale for several items might be discussed together.	5
Researcher characteristics and reflexivity	#6	Researchers' characteristics that may influence the research, including personal attributes, qualifications / experience, relationship with participants, assumptions and / or presuppositions; potential or actual interaction between researchers' characteristics and the research questions, approach, methods, results and / or transferability	7
Context	#7	Setting / site and salient contextual factors; rationale	5
Sampling strategy	#8	How and why research participants, documents, or events were selected; criteria for deciding when no further sampling was necessary (e.g. sampling saturation); rationale	5, 17
Ethical issues pertaining to human subjects	#9	Documentation of approval by an appropriate ethics review board and participant consent, or explanation for lack thereof; other confidentiality and data security issues	7
Data collection methods	#10	Types of data collected; details of data collection procedures including (as appropriate) start and stop dates of data collection and analysis, iterative process, triangulation of sources / methods, and modification of procedures in response to evolving study findings; rationale	6

For peer review only

Data collection instruments and technologies	#11	Description of instruments (e.g. interview guides, questionnaires) and devices (e.g. audio recorders) used for data collection; if / how the instruments(s) changed over the course of the study	6
Units of study	#12	Number and relevant characteristics of participants, documents, or events included in the study; level of participation (could be reported in results)	5-6
Data processing	#13	Methods for processing data prior to and during analysis, including transcription, data entry, data management and security, verification of data integrity, data coding, and anonymisation / deidentification of excerpts	6-7
Data analysis	#14	Process by which inferences, themes, etc. were identified and developed, including the researchers involved in data analysis; usually references a specific paradigm or approach; rationale	6-7
Techniques to enhance trustworthiness	#15	Techniques to enhance trustworthiness and credibility of data analysis (e.g. member checking, audit trail, triangulation); rationale	6-7
**Results/findings**			
Syntheses and interpretation	#16	Main findings (e.g. interpretations, inferences, and themes); might include development of a theory or model, or integration with prior research or theory	7-14
Links to empirical data	#17	Evidence (e.g. quotes, field notes, text excerpts, photographs) to substantiate analytic findings	7-13
**Discussion**			
Intergration with prior work, implications, transferability and contribution(s) to the field	#18	Short summary of main findings; explanation of how findings and conclusions connect to, support, elaborate on, or challenge conclusions of earlier scholarship; discussion of scope of application / generalizability; identification of unique contributions(s) to scholarship in a discipline or field	14-16
Limitations	#19	Trustworthiness and limitations of findings	17

For peer review only

Other

Conflicts of interest	#20	Potential sources of influence of perceived influence on study conduct and conclusions; how these were managed	19
Funding	#21	Sources of funding and other support; role of funders in data collection, interpretation and reporting	18

The SRQR checklist is distributed with permission of Wolters Kluwer © 2014 by the Association of American Medical Colleges. This checklist was completed on 14. June 2019 using https://www.goodreports.org/, a tool made by the EQUATOR Network in collaboration with Penelope.ai

For peer review only

BMJ Open

Suffering out of sight but not out of mind – interpreting persons' experiences of sick leave due to chronic pain in a community setting: a qualitative study

Journal:	*BMJ Open*
Manuscript ID	bmjopen-2022-066617.R1
Article Type:	Original research
Date Submitted by the Author:	20-Feb-2023
Complete List of Authors:	Lundin, Åse; University of Gothenburg Institute of Health and Care Sciences, Sahlgrenska Academy, University of Gothenburg; University of Gothenburg Centre for Person-Centred Care, Sahlgrenska Academy, University of Gothenburg<br>Ekman, Inger; University of Gothenburg Institute of Health and Care Sciences, Sahlgrenska Academy, University of Gothenburg; University of Gothenburg Centre for Person-Centred Care, Sahlgrenska Academy, University of Gothenburg<br>Wallström, Sara; University of Gothenburg Institute of Health and Care Sciences, Sahlgrenska Academy, University of Gothenburg; University of Gothenburg Centre for Person-Centred Care, Sahlgrenska Academy, University of Gothenburg<br>Andréll, Paulin; Sahlgrenska University Hospital Ostra Hospital, Department of Anaesthesiology and Intensive Care Medicine/Pain Centre; University of Gothenburg Institute of Clinical Sciences, Department of Anaesthesiology and Intensive Care Medicine, Sahlgrenska Academy<br>Lundberg, Mari; Sophiahemmet University, Department of Health Promoting Science; University of Gothenburg Centre for Person-Centred Care, Sahlgrenska Academy, University of Gothenburg
**Primary Subject Heading**:	Rehabilitation medicine
Secondary Subject Heading:	Qualitative research, Public health
Keywords:	QUALITATIVE RESEARCH, PAIN MANAGEMENT, PUBLIC HEALTH, REHABILITATION MEDICINE

SCHOLARONE™
Manuscripts

I, the Submitting Author has the right to grant and does grant on behalf of all authors of the Work (as defined in the below author licence), an exclusive licence and/or a non-exclusive licence for contributions from authors who are: i) UK Crown employees; ii) where BMJ has agreed a CC-BY licence shall apply, and/or iii) in accordance with the terms applicable for US Federal Government officers or employees acting as part of their official duties; on a worldwide, perpetual, irrevocable, royalty-free basis to BMJ Publishing Group Ltd ("BMJ") its licensees and where the relevant Journal is co-owned by BMJ to the co-owners of the Journal, to publish the Work in this journal and any other BMJ products and to exploit all rights, as set out in our licence.

The Submitting Author accepts and understands that any supply made under these terms is made by BMJ to the Submitting Author unless you are acting as an employee on behalf of your employer or a postgraduate student of an affiliated institution which is paying any applicable article publishing charge ("APC") for Open Access articles. Where the Submitting Author wishes to make the Work available on an Open Access basis (and intends to pay the relevant APC), the terms of reuse of such Open Access shall be governed by a Creative Commons licence – details of these licences and which Creative Commons licence will apply to this Work are set out in our licence referred to above.

Other than as permitted in any relevant BMJ Author's Self Archiving Policies, I confirm this Work has not been accepted for publication elsewhere, is not being considered for publication elsewhere and does not duplicate material already published. I confirm all authors consent to publication of this Work and authorise the granting of this licence.

For peer review only

Suffering out of sight but not out of mind – interpreting persons' experiences of sick leave due to chronic pain in a community setting: a qualitative study

Word count, excluding title page, abstract, declarations, references and tables: **5084**

Åse Lundin; corresponding author (Orcid ID 0000-0001-9980-7653)
ase.lundin@gu.se
Arvid Wallgrens backe, hus 1
Box 457, 405 30 Göteborg, Sweden

- Institute of Health and Care Sciences, Sahlgrenska Academy, University of Gothenburg, Gothenburg, Sweden
- University of Gothenburg Centre for Person-Centred Care (GPCC), Sahlgrenska Academy, University of Gothenburg, Gothenburg, Sweden

Inger Ekman, Orcid ID: 0000-0002-5559-5203

- Institute of Health and Care Sciences, Sahlgrenska Academy, University of Gothenburg, Gothenburg, Sweden
- University of Gothenburg Centre for Person-Centred Care (GPCC), Sahlgrenska Academy, University of Gothenburg, Gothenburg, Sweden

Sara Wallström: Orcid ID:0000-0001-7579-4974

- Institute of Health and Care Sciences, Sahlgrenska Academy, University of Gothenburg, Gothenburg, Sweden
- University of Gothenburg Centre for Person-Centred Care (GPCC), Sahlgrenska Academy, University of Gothenburg, Gothenburg, Sweden
- Forensic psychiatric department, Sahlgrenska University Hospital, Gothenburg, Sweden

Paulin Andréll: Orcid ID 0000-0001-6941-6096

- Region Västra Götaland, Sahlgrenska University Hospital/Östra, Department of Anaesthesiology and Intensive Care Medicine/Pain Centre, Gothenburg, Sweden
- Department of Anaesthesiology and Intensive Care Medicine, Institute of Clinical Sciences at Sahlgrenska Academy, University of Gothenburg, Gothenburg, Sweden

Mari Lundberg: Orcid ID 0000-0001-7123-0362

- Department of Health Promoting Science, Sophiahemmet University, Stockholm, Sweden

For peer review only - http://bmjopen.bmj.com/site/about/guidelines.xhtml

For peer review only

- University of Gothenburg Centre for Person-Centred Care (GPCC), Sahlgrenska Academy, University of Gothenburg, Gothenburg, Sweden

Abstract

Objective: Chronic pain is a complex health problem affecting about one-fifth of the European population. It is a leading cause of years lived with disability worldwide with serious personal, relational, and socio-economic consequences. Chronic pain and sick leave adversely affect health and quality of life. Thus, understanding this phenomenon is essential to reduce suffering, understand the need for support, and promote a rapid return to work and an active lifestyle. This study aimed to describe and interpret persons' experiences of being on sick leave due to chronic pain.

Design: A qualitative study with semi-structured interviews analysed using a phenomenological hermeneutic approach.

Setting: The participants were recruited from a community setting in Sweden.

Participants: Fourteen participants (12 women) with experiences of part-time or full-time sick leave from work due to chronic pain were included in the study.

Findings: *Suffering out of sight but not out of mind* was the main theme of the qualitative analysis. This theme means that the participants' constant suffering was invisible to others, causing them to feel they were not being justly treated in society. Feeling overlooked led to a continuous struggle for recognition and the participants' identities and their trust in themselves and their bodies was challenged. However, our study also revealed a nuanced understanding of the experiences of sick leave due to chronic pain where the participants learned important lessons including coping strategies and re-evaluated priorities.

For peer review only - http://bmjopen.bmj.com/site/about/guidelines.xhtml

Conclusions: Being on sick leave due to chronic pain threatens a person's integrity and leads to great suffering. A deepened understanding of the meaning of the personal impact of being on sick leave due to chronic pain provides important considerations for care and support of these persons and highlights the importance of feeling acknowledged and met with justice in their encounters with others.

Keywords:

Chronic pain, sick leave, qualitative research, phenomenological hermeneutics, lived experiences

Strengths and limitations of this study

- Qualitative research is important in enlightening the personal experiences of pain and its consequences, uncovering information often not accessible through other methods
- Exploring meanings of lived experiences can help us understand and improve our practices in health care
- Studies using interpretative qualitative methods is encouraged within the field of chronic pain and addresses a gap in the existing literature
- In this study only two out of 14 participants were men, and this distribution might have affected the findings

Introduction

Chronic pain has reached alarming levels, particularly among older adults, and is one of the leading causes of years lived with disability (1). Pain of moderate to severe intensity affects 19% of the European population (2). The socio-economic burden of chronic pain is substantial, mainly for working-age people, with the bulk of indirect costs related to absence

For peer review only

For peer review only - http://bmjopen.bmj.com/site/about/guidelines.xhtml

from the workforce (3). In the US the total costs associated with chronic pain in adults range from 560–635 billion USD, exceeding the annual costs estimated for heart disease (309 billion USD), cancer (243 billion USD) and diabetes (188 billion USD) (4). In Sweden, the cost of a diagnosis related to chronic pain was estimated to be 32 billion euros per year (3). Chronic pain has a significant impact on quality of life. Suffering from chronic pain is closely associated with depression (5), increased suicide rates (6), premature mortality and reduced life expectancy (7, 8). Because a key feature of pain is that it is a "*personal experience that is influenced to varying degrees by biological, psychological, and social factors"* (9), a person's experience or report of pain is of particular interest and should be taken seriously (10). Qualitative research is growing in the field of pain, exposing aspects of experiencing pain that is not accessible through other approaches (11). Previous studies of people's experiences with chronic pain revealed feelings of restriction, loss and limitation of daily life (12, 13), loneliness and silent suffering (14). These studies also showed that chronic pain deeply affected relationships, family life, and work (13). In addition, being on sick leave negatively impacts a person's life. Research on the experiences of being absent from work due to illness has been done in different populations and for different health conditions, revealing negative experiences, including loss of identity (15) social exclusion and a struggle to adapt to reduced work capacity (16). Being in the sick leave process also led to feelings of uncertainty and insecurity (17), stigma, helplessness, and a loss of independence (18).

Despite implementation of evidence-based practices, such as interdisciplinary rehabilitation, many persons with chronic pain do not return to work (19). Qualitative pain research has often focused on the experiences of barriers and facilitators of return to work for people with chronic pain (20, 21). However, there are limited research on the personal experiences of being in the sick leave process due to chronic pain. Some studies reflect on immigrant women's experiences of long-term sick leave due to chronic pain. The studies found these

For peer review only - http://bmjopen.bmj.com/site/about/guidelines.xhtml

women felt excluded and rejected (22), struggling with an invisible physical ailment (23). Other studies have explored the phenomenon on a descriptive level (24). Yet, researchers have been encouraged to go beyond description and towards interpretative engagement while performing qualitative analyses of pain (11). In line with this approach, we sought to understand the essential meaning of sick leave due to chronic pain. A deepened understanding of the personal experiences of the phenomenon is vital in order to reduce suffering, better understand their needs and facilitate a return to working life.

This study aimed to describe and interpret persons' experiences of being on sick leave due to chronic pain.

Method

The authors followed the Standards for Reporting Qualitative Research (SRQR) guidelines (25) to ensure the trustworthiness of the findings.

Design

In this study we chose a qualitative interview design to explore people's experiences of living with chronic pain and sick leave. Data in the form of transcripts of semi-structured interviews were analyzed using phenomenological hermeneutics inspired by Ricoeur (26) and developed by Lindseth and Norberg (27). The method has been used in numerous health and care studies (e.g., (28-30). The method aims to understand the meaning of the participants' lived experiences by interpreting their narratives.

Participants and setting

The inclusion criteria for this study were: having experience of being on sick leave part-time or full-time due to chronic pain (duration of pain >3 months), speaking Swedish or English

For peer review only - http://bmjopen.bmj.com/site/about/guidelines.xhtml

and being able and willing to participate in an interview. Recommended target size in qualitative interview studies vary, phenomenological research often aim to involve 5 to 25 persons who have experienced the phenomenon (31). ÅL, SW and IE regularly evaluated the richness of the collected data in regard to the research question during the conduction of the interviews to help determine when a sufficient number of participants had been included.

The participants were recruited through patient organizations and associations that focus on pain or pain-related conditions. A request for participation was sent out through their e-mail, newsletter or Facebook pages. The participants were able to choose the location of the interview and if they wanted to meet face-to-face or through digital channels.

Data collection

Data were collected through individual semi-structured in-depth interviews that were audio-recorded and transcribed verbatim. All interviews and transcripts were confidential using numerical coding, and precautions were taken to safeguard the identity of the participants.

An interview guide was used (see supplemental material). All interviews opened with the question *"I understand you have been living in pain for a long time. Would you tell me about what this time has been like for you?"*. Follow-up and probing questions such as "*can you give me an example of that?"* and *"how did that make you feel?"* were used to encourage further narration.

Being semi-structured interviews, this guide was not used as a strict list of questions, but rather as help to move the conversation forward and make sure the research questions were covered during the interview. Some participants narrated more freely and other participants needed more guidance to address the research questions.

Analysis and interpretation of data

Phenomenological hermeneutics comprises three interwoven parts: naïve reading, structural analysis and interpretation of the whole (27). During the naïve reading, the text was read repeatedly to grasp its meaning as a whole. In the structural analysis the text related to the aim of the study was divided into meaning units that were then abstracted and formed into subthemes, themes and a main theme. A qualitative data analysis software, QSR NVivo R1,

For peer review only - http://bmjopen.bmj.com/site/about/guidelines.xhtml

was used in the structural analysis. The analysis was a continuous process moving between the parts and meaning units of the text and the overall understanding. ÅL, ML and IE met regularly during this process to discuss the collected data and the emergence of subthemes, themes and main theme to highlight different perspectives, add breadth and nuances but also to ensure a congruent reflection and understanding of the narratives. All parts of the text were considered, but the parts not relevant to the research question were not included in the analysis. During the final part of the analysis, the text was reread, and the initial naïve reading, the structural analysis and the authors' preunderstandings were combined to interpret the text's overall meaning. We used research related to the area, theoretical literature and discussions among the authors to gain a clearer and deeper understanding of the text.

Research characteristics and reflexivity

ÅL is a physiotherapist with several years of clinical experience in primary health care. The author has frequently encountered patients seeking care for chronic pain who cannot work due to their condition. She has previously done qualitative research but not in areas related to pain. Of the co-authors, two have long experience in research on pain (ML and PA) and three have extensive experience working with qualitative analyses (IE, ML, and SW).

Ethical considerations

Written consent was obtained from each participant. The study was approved by the Swedish Ethical Review Authority DNR 2020-02491 and complied with the principles in the Declaration of Helsinki (32).

Patient and public involvement

Before the study was undertaken, several patient associations were contacted to help recruit participants. These patient organizations and their channels will also be used to disseminate the study results to reach a wider audience beyond the academic community.

Findings

Out of the 18 people contacted the first author (ÅL) regarding participation, three decided not to go through with an interview and one did not show up for the booked interview. Fourteen participants (12 women and 2 men) were included in the study and went on to be interviewed. Participant characteristics are described in Table 1.

For peer review only - http://bmjopen.bmj.com/site/about/guidelines.xhtml

Table 1

**Participant characteristics**	**(n = 14)**
Men/Women	2/12
Mean age, years (range)	49 (23-80)
Current work status	
Full-time sick leave	4
Part-time sick leave	5
Returned to work	4
Retired	1
Highest completed education	
Primary school (9 years)	2
High School	3
Other upper secondary education	4
University degree	5
Duration of pain	
1-3 years	2
4-9 years	1
>10 years	11

The location and causes of the chronic pain varied among the participants and low back pain, neck pain, rheumatism, fibromyalgia, shoulder pain, arthritis, endometriosis, joint pain due to Ehler Danlos syndrome as well as whiplash injury and spinal injury was represented among the participants.

The interviews were conducted in 2021 by ÅL and lasted from 41-90 minutes. Ten interviews were performed through digital channels; two were in the participants' homes and two on the university's premises.

Naïve reading

The naïve reading of the text indicated that living with chronic pain and being on sick leave significantly impacted the participants' lives and how they identified themselves. The feelings of exclusion, loss and unfamiliarity permeated the naïve understanding. The participants struggled to find ways to manage a condition experienced as invisible to others and hindered participation in daily activities and work.

For peer review only - http://bmjopen.bmj.com/site/about/guidelines.xhtml

Structural analysis

The structural analyses partly validated our first impressions. These analyses also helped nuance and highlight other parts of the phenomenon, mainly regarding dependency and relations with institutions, understandings of the phenomenon that went unnoticed in the first part of the analysis.

An overview of the main theme, themes and subthemes from the structural analysis is given in Table 2 and further described below.

Table 2
Overview of the main theme, themes and subthemes

Suffering out of sight but not out of mind		
**An identity under threat**	**Caught in a foreign body**	**Navigating uncharted waters**
Becoming dependent<br>Being excluded<br>Feeling flawed	Losing bodily trust<br>Feeling constantly tired	Constantly chasing recognition<br>A journey towards unexpected lessons

An identity under threat

Being on sick leave due to chronic pain affected and threatened the participants' views of themselves and their identity, significantly impacting their relationships with others. This theme was expressed in three subthemes: Becoming dependent, Being excluded and Feeling flawed.

1. Becoming dependent

The participants described no longer recognizing themselves concerning their capabilities. They had previously viewed themselves as independent and able to care and fend for themselves. However, being on sick leave due to pain forced them to become increasingly

For peer review only - http://bmjopen.bmj.com/site/about/guidelines.xhtml

dependent on others. Becoming dependent involved having to rely on help from family and friends, outsource physical household chores and become economically reliant on their partners to pay for their dwelling or provide for the children. Participants experienced frustration from relying on authorities' benefits and goodwill regarding their work status and sick leave payments. Decisions were often out of the participants' control, causing distress and a feeling of worthlessness and powerlessness. One participant who was denied access to sick leave benefits said,

> *In the end, you feel like you are just a burden to your husband [crying]. I have no income to get by myself. I cannot earn a living. And if he cannot service or help me or fix the car, then we have to sell it because I can't do it. # 4*

2. Being excluded

The presence of pain and being on sick leave led to being left out of the workforce and losing value as an employee and person. The participants previously felt pride in being hard workers and wanted to return to work and be a part of the social life and the routines they had at the workplace that made them feel meaningful and appreciated. Having to go on sick leave was experienced as being imprisoned or punished and a struggle of being left behind.

> *Being at home it…I think it is the worst punishment. The days…the days are long when you are healthy so you can imagine when you are sick and restricted and…and being alone in the house and my husband is working and my daughter is at school and then you think a lot of negative thoughts and you have to occupy the brain, I guess. #14*

For peer review only - http://bmjopen.bmj.com/site/about/guidelines.xhtml

3. Feeling flawed

Because the participants lived with pain and could not work, they felt flawed as persons, parents, and participants in daily life. They struggled with a loss of engagement, feeling unhappy, increased anger, short temper towards family members, and other personality changes. They often had to decline participation in social activities or hobbies against their wishes. It affected their relationships with family and friends, creating grief, largely concerning being unable to be there for their friends, spouse, or children as they used to be.

> *But you still feel that it's like you are not sufficiently there. So, you also have a constant feeling of guilt that "no, mommy isn't able to…". #6*

Caught in a foreign body

Suffering from chronic pain meant not recognizing the body as the familiar place it used to be, creating conflicts that made it difficult to cope with daily and working life. This theme was expressed in two subthemes: Losing bodily trust and Feeling constantly tired.

1. Losing bodily trust

Experiencing distressing physical symptoms combined with intensive and unreliable pain fluctuations was very tiresome for the participants. Not knowing how their body would function or react from day to day created a loss of trust and a feeling that their body was not the safe and trusted home it used to be. One participant described it as always wearing a heavy diving suit, interpreted as a conflict between what the participant wanted and what the body could do. Another participant who worked as a caretaker described how the sometimes

For peer review only - http://bmjopen.bmj.com/site/about/guidelines.xhtml

rapid onset of pain made her feel insecure and losing trust in her body while helping and interacting with the elderly in their homes.

Then I would make supper and help them into their nightclothes, and then you ought to…some of them who were in wheelchairs would have toilet visits and then do many things. And I drop pots and cups on sinks, and it banged and these older people, 80-90 years old, were terrified! And I got so much pain that I could barely lift this pot back up again, or the cup or… I mean, I would have hot, both hot water and stuff. I'm terrified that I would drop an old…/… /And then I did not want to go back there when I know that I do not, that I cannot hold. When I cannot even grab a cup, how can I manage to hold a whole person? # 4

2. Feeling constantly tired

The participants experienced that fatigue and the inability to sleep were a greater disturbance in their daily lives and their ability to work than the pain itself. The little energy possibly gained through the night was quickly drained the next day when coping with the fatigue associated with the pain. Sleepless nights, the side effects of sleep medication, and not feeling rested were associated with great stress and suffering. Being on sick leave and not having to get up for work opened up the possibility to rest a little during the day instead of the night, which was interpreted as a small window of opportunity to find relief.

Because it's, it's, it's actually the worst, really. The pain, you can do something about it, you can, it can be alleviated...but the fatigue cannot be alleviated in the same way. #10

Navigating uncharted waters

For peer review only - http://bmjopen.bmj.com/site/about/guidelines.xhtml

Living in pain and being unable to work was interpreted as navigating through the unfamiliar, a route filled with unpleasantness and uncertainties (but sometimes helpful surprises). This theme was expressed in two subthemes: Constantly chasing recognition and A journey towards unexpected lessons.

1. Constantly chasing recognition

Throughout the narratives, it became clear that living with pain meant facing mistrust from most parts of society. Pain was experienced as a hidden disease by the participants. An invisible condition made them feel invariably questioned, especially in contact with the authorities, the healthcare system, and their workplace. They felt that they were perceived as complainers, being lazy, and making up or exaggerating their symptoms. It was interpreted as a constant and never-ending pursuit to be believed and have their suffering recognized and taken seriously. One participant described not being met with the support needed early enough from the health care system and how that might have prolonged her sick leave;

> *If the scenario had been such that the health care system actually took care of me and had helped me, then I wouldn't have had... even though I will always have problems because I'm chronically ill, I will always have problems, but it hadn't been that noticeable. It would not have been so extensive and so often and so strong. Which would have meant that I could have been able to handle it and I would have been able to work # 9*

Instead, the participants continuously fought to receive a diagnosis that could be shown through objective testing to explain their pain and validate them in both getting access to treatment and their leave of absence from work.

> *It does not matter what I say because it feels like they see my smile and that I am active and walking that "well, but then she is not so ill." And if I had gone back to*

For peer review only - http://bmjopen.bmj.com/site/about/guidelines.xhtml

*work "but then you are not so ill," and it starts all over again!/…./Just because it is not visible on a damn test…That you couldn't just, you know you can connect cars to computers, couldn't you get a frigging body scan and *swoosh* where it lights up red, she's in pain there! # 3*

2. A journey towards unexpected lessons

The participants were forced to find different coping strategies and new routines because of the profound fatigue and constant pain. This endeavour was interpreted as a bumpy journey with uncertainties and worries, especially regarding the future and how their life and work will be if their condition deteriorates. The participants' experiences of living with pain were not all undesirable in that some had learned important life lessons. They gained new insights about the importance of not pushing oneself too hard, that rest is imperative, and that it is not beneficial to let work dominate life. They identified abilities and resources within themselves that they had not previously seen that helped them cope with their new situation. They also re-evaluated their priorities regarding relationships and family and the importance of appreciating the small things in life.

I think I'm better at thinking that the small things are good and pleasant. It may be enough that I go for a walk in the woods and look at the water. You will still be all right, or better. In a way that I didn't …I didn't see the qualities of the little things in life quite the same way. Maybe it was the bigger stuff that was more important. The rest was mostly just everyday things. I do not think that way anymore; instead, I believe that there are small things that are important after all. #8

For peer review only - http://bmjopen.bmj.com/site/about/guidelines.xhtml

Interpretation of the whole

The naïve reading, the structural analysis, and the authors' preunderstandings were combined to interpret the overall meaning of the text material. The interpretation was inspired by Ricoeur's philosophy and ethical intentions of *"aiming at a good life with and for others in just institutions"* (33), emphasizing that ethics is prioritized over morality or norms. Ricoeur's philosophy on just institutions is of particular interest to this study as it is essential that people feel justly treated when in contact with institutions (34) such as the healthcare system, the authorities, or the workplace. With pain being experienced as a hidden and questionable illness, there was an incessant pursuit to have their life situation validated and recognized by others. We interpret this as being about justice, a feeling that they were not receiving or being met with the justice they deserved. During their sick leave, the participants' identities were threatened and challenged by feelings of flaws, exclusion, and dependency. They felt they were caught in their bodies, which they experienced as foreign, not entirely their own. The themes were interpreted as all being connected by being processes that were invisible to others, to institutions, but still brought much suffering to the participants. A suffering that was exacerbated by the feelings of not being treated fairly. Although their struggles were hidden from the outside world, they were real to the participants, affecting all aspects of life. Based on this, the meaning of living with and being on sick leave due to chronic pain was interpreted as *Suffering out of sight but not out of mind,* which could be regarded as the main theme. It is a play on the idiom "out of sight, out of mind," meaning that you soon forget people (or things) that are no longer visible or present. For the participants, however, the pain and suffering were permanent and real.

Discussion

For peer review only - http://bmjopen.bmj.com/site/about/guidelines.xhtml

The main findings of this study are that the participants experienced their suffering as invisible to others, causing them to feel they were not being justly treated in society and constantly had to struggle for recognition. Moreover, being on sick leave due to chronic pain challenged the participants' identities and their trust in themselves and their bodies. Contrary to previous research, which has mainly highlighted the hardships and adversities of sick leave (16, 17, 22, 23), our study revealed a nuanced understanding of the experiences of being on sick leave due to chronic pain. Despite their difficulties, and sometimes to their surprise, the participants felt that this situation had taught them new things about themselves, forced them to develop new coping-strategies and given them opportunities to embrace other aspects of life. Research has shown that the presence of supportive experiences (e.g., meeting respectful health care professionals) is important to those on sick leave (18) and that possessing problem-solving coping strategies significantly reduces sick leave (35). In our study we also interpreted the participants' learning experiences as something that stretched over a broader context and brought added value to them regarding relationships, family, and having a positive approach to life. They were insights and understanding that would not have been reached without the participants facing the process of illness and sick leave. Our findings are important because if the participants had learned these lessons (e.g., coping strategies) earlier, sick leave might have been shortened or prevented. The participants in our study also explained that they might have continued working if their struggles had been recognized at the workplace or by the healthcare system from the start. We interpret this inadequacy as lacking the institutional support they needed, and looking at it in regard to Ricoeur, not met by what would be considered a just institution.

The experience of being in a foreign body when suffering from poor health has been described in other studies. In illness the body can be perceived as unhomelike and can be seen as a "broken tool" that is no longer in control, resulting in a loss of command over one's

For peer review only - http://bmjopen.bmj.com/site/about/guidelines.xhtml

own body (36). Suffering from chronic pain could also be interpreted as experiencing the body as an obstruction and being confronted with having a body that feels different than when healthy (37). The participants in our study described how they were affected by several neurophysiological changes due to pain including fatigue, stress and mood changes such as increased anger. According to our clinical experiences this might represent a lack of knowledge of the dialectical relationship between the body and psychological or behavioural changes. Increasing the understanding of the bidirectional relationship between the mind and the body and how this interaction affects the pain experience is an important part in pain rehabilitation. Pain neuroscience education has been shown to increase this knowledge and reduce disability among patients suffering from chronic pain (38). Our study also found that the loss of familiarity with the body when suffering from pain hindered the participants' return to work. The ability to return to work was interpreted as not only a question of the physical strength needed but also trust in their capabilities and feeling safe in their bodies in the context of the workplace. As shown in previous research, the experience of moving while in persistent pain implies more than just physical restraints. It challenges and threatens a person's entire lifeworld and existence (39). In the words of Merleau-Ponty, our lived body is our access to or *"a vehicle of being in the world"* (40) and when our body changes, or in this case, where the participants felt trapped in an unknown body due to pain, it alters the way the world is perceived.

Our participants viewed their identities as challenged or under threat while on sick leave due to pain. This feeling is consistent with previous findings on the sick leave process due to other health conditions, expressed as being reduced as a person and not recognizing oneself (15) or experiencing a changed self-perception (18). Our participants described a strong connection between their identity and their work. Losing contact with the workplace meant losing who they were as productive persons. The authenticity of pain conditions as a cause of

For peer review only - http://bmjopen.bmj.com/site/about/guidelines.xhtml

work absence has been questioned by some employers, suggesting that workers are "lazy" (21). In contrast, our study shows a strong desire to return to work and the appreciation they felt was linked to being part of the workforce.

We interpreted the experiences of constantly chasing recognition while facing distrust and suspicion from others as not being met by just institutions. The meaning of a just institution is that each person when in contact with for example the health care system senses justice on a fundamental level, i.e., is treated well and considered equal partners in the care process (34). A just institution is a crucial part of person-centred care and allows persons to express their needs and abilities and even offer a sense of partnership (34). In our study, however, the participants described feeling subservient and helpless, with their lives dependent on the goodwill of institutional gatekeepers. Previous research on sick leave has observed similar experiences, i.e. instead of a partnership, the rules and regulations make the stakeholders become counterparts, leaving little flexibility for the person (15). But being on sick leave due to chronic pain was also interpreted as different from other health issues, mainly because of its invisibility and the lack of objective testing of pain symptoms. A social stigma is attached to chronic pain (41) and in our study one participant wondered whether the workplace or authorities would have looked at her and her sick leave in a different light if she had cancer rather than a chronic pain condition? In 2018 the International Classification of Diseases was revised (ICD-11) and for the first time includes a coding system for chronic pain (42). Acknowledging and recognizing chronic pain as a disease in itself and not just a symptom, is an opportunity to improve treatment as well as our understanding of chronic pain and its management (42). Another benefit is that this new classification may potentially shift the focus from finding a cause to instead manage the impact of the chronic pain for the patient, for example through multimodal treatments (42). The participants in this study often fought hard to receive a diagnosis and to be able to objectively prove to the health care professionals

For peer review only - http://bmjopen.bmj.com/site/about/guidelines.xhtml

that they were in pain to receive the right help. The new ICD-11 can hopefully be part of acknowledging the importance of understanding in rehabilitation and in the process of returning to work, as well as potentially reduce stigma by increasing both the legitimacy and the visibility of chronic pain.

Methodological considerations

In the current study only two out of 14 participants were men. This skewed distribution might partly be explained by research showing that women report a higher prevalence of chronic pain (43) but could also be influenced by other factors (e.g., women being overrepresented or more active in the patient organizations used here to recruit participants). The study also explored the 14 participants' narratives, and their experiences might not apply to other people on sick leave due to other chronic pain conditions. However, qualitative research does not argue that the findings are universal and generalizable; rather, it explores possible meanings of lived experiences to understand and improve our practices in health care (27).

Sample size in qualitative studies is often a subject of discussion, where no power calculations can determine the sample size needed (44). In phenomenological research sample sizes of between 5 and 25 persons are commonly suggested (31). But determining sample size and when no further sampling is needed is a matter of judgment and experience in evaluating the quality of already collected data to the aims and methods used (44). During the inclusion process and inspection of the collected interview data, the material was discussed within the research group and deemed to contain rich and varied narratives of the experiences, sufficient to answer the research question using the phenomenological hermeneutic method.

For peer review only - http://bmjopen.bmj.com/site/about/guidelines.xhtml

Another potential limitation is related to the COVID-19 pandemic restrictions. Because of the restrictions, most interviews were performed digitally, which could have affected the interactions and dynamics during the interviews compared to face-to-face encounters. A recent review showed that digital interviews could adversely affect the interviews, including experiencing technical issues or difficulties reading visual cues or body language. The review also found that richer data could be extracted through a digital approach when investigating sensitive topics (e.g., health issues), most likely because the participants felt more in control of the situation and the setting and thus felt more relaxed (45).

Conclusions, clinical implications, and future research

This study underscores the experiences of persons on sick leave due to chronic pain, where feelings of being invisible and overlooked often dominate. They do not feel that they are met with justice in their encounters with their surroundings, creating existential concerns and threatening their identities, bodies, and roles to others. In contrast to previous research which have mainly high-lighted the adversities of being in the sick leave process, our study revealed a nuanced understanding of the participants' learning experiences of sick leave due to chronic pain. A deepened understanding of the essential meaning of these experiences and gaining new insights of the personal impact of being on sick leave due to chronic pain provides important considerations for care and support of these persons and means for a joint planning of rehabilitation towards a return to work. It is important to understand their lifeworld in order to be able to raise and acknowledge the issues of existential threat, stigma, invisibility and feelings of injustice that these persons experience in encounters with others. Suggestions for future research include further looking into what factors might play a role in supporting

For peer review only - http://bmjopen.bmj.com/site/about/guidelines.xhtml

persons on sick leave due to chronic pain in returning to work as well as exploring the impact of the new ICD-classification for these patients.

Declarations

- **Ethics approval**

  The study was approved by the Swedish Ethical Review Authority DNR 2020-02491 and conformed to the Declaration of Helsinki (32). Written informed consent was obtained from each participant at the start of the study.

- **Funding**

  This work was supported by FORTE grant number 2019-00718 and The University of Gothenburg Centre for Person-Centred Care (GPCC) grant number N/A.

- **Contributorship**

  All authors participated in the conception and design of the study. ÅL recruited participants, conducted and transcribed the interviews and performed the analysis and interpretation of data with critical input from IE and ML. The manuscript was drafted by ÅL and critically revised and edited by IE, ML, SW and PA. All authors read and approved the final version of the manuscript and agree to be accountable for all aspects of the work in ensuring that questions related to the accuracy or integrity of any part of the work are appropriately investigated and resolved.

- **Competing interests**

  None declared

- **Data sharing statement**

  The interview data are not publicly available.

- **Patient consent form**

For peer review only - http://bmjopen.bmj.com/site/about/guidelines.xhtml

Not required

- **Acknowledgments**

We acknowledge the participants for taking the time and effort to share their narratives and experiences. Without them, this study would not have been possible.

References

1. Rice ASC, Smith BH, Blyth FM. Pain and the global burden of disease. PAIN. 2016;157(4).
2. Breivik H, Collett B, Ventafridda V, Cohen R, Gallacher D. Survey of chronic pain in Europe: prevalence, impact on daily life, and treatment. European journal of pain (London, England). 2006;10(4):287-333.
3. Gustavsson A, Bjorkman J, Ljungcrantz C, Rhodin A, Rivano-Fischer M, Sjolund K-F, et al. Socio-economic burden of patients with a diagnosis related to chronic pain – Register data of 840,000 Swedish patients. 2012;16(2):289-99.
4. Gaskin DJ, Richard P. The economic costs of pain in the United States. The journal of pain. 2012;13(8):715-24.
5. Bair MJ, Robinson RL, Katon W, Kroenke K. Depression and Pain Comorbidity: A Literature Review. Archives of Internal Medicine. 2003;163(20):2433-45.
6. Tang NKY, Crane C. Suicidality in chronic pain: a review of the prevalence, risk factors and psychological links. Psychological Medicine. 2006;36(5):575-86.
7. Torrance N, Elliott AM, Lee AJ, Smith BH. Severe chronic pain is associated with increased 10 year mortality. A cohort record linkage study. 2010;14(4):380-6.
8. Smith D, Wilkie R, Uthman O, Jordan JL, McBeth J. Chronic Pain and Mortality: A Systematic Review. PLOS ONE. 2014;9(6):e99048.
9. Raja SN, Carr DB, Cohen M, Finnerup NB, Flor H, Gibson S, et al. The revised International Association for the Study of Pain definition of pain: concepts, challenges, and compromises. 2020;161(9):1976-82.
10. Cohen SP, Vase L, Hooten WM. Chronic pain: an update on burden, best practices, and new advances. The Lancet. 2021;397(10289):2082-97.
11. Osborn M, Rodham K. Insights into Pain: A Review of Qualitative Research. Rev Pain. 2010;4(1):2-7.
12. Carson MG, Mitchell GJ. The experience of living with persistent pain. Journal of advanced nursing. 1998;28(6):1242-8.
13. De Souza L, Oliver Frank A. Patients' experiences of the impact of chronic back pain on family life and work. Disability and Rehabilitation. 2011;33(4):310-8.
14. Gillsjö C, Nässén K, Berglund M. Suffering in silence: a qualitative study of older adults' experiences of living with long-term musculoskeletal pain at home. European journal of ageing. 2021;18(1):55-63.
15. Andersson C, Mårtensson L. Women's experiences of being in the sick leave process. Scandinavian journal of occupational therapy. 2021;28(6):488-97.

For peer review only - http://bmjopen.bmj.com/site/about/guidelines.xhtml

16. Severinsen A, Midtgaard J, Backhausen MG, Broberg L, Hegaard HK. Pregnant women's experiences with sick leave caused by low back pain. A qualitative study. Work (Reading, Mass). 2019;64(2):271-81.
17. Lindbäck C, Nordgren L. To be on sick-leave due to heart failure: a qualitative perspective. Disabil Rehabil. 2015;37(19):1732-8.
18. Lännerström L, Wallman T, Holmström IK. Losing independence – the lived experience of being long-term sick-listed. BMC Public Health. 2013;13(1):745.
19. LoMartire R, Björk M, Dahlström Ö, Constan L, Frumento P, Vixner L, et al. The value of interdisciplinary treatment for sickness absence in chronic pain: A nationwide register-based cohort study. 2021;25(10):2190-201.
20. Wainwright E, Wainwright D, Keogh E, Eccleston C. Return to work with chronic pain: employers' and employees' views. Occupational Medicine. 2013;63(7):501-6.
21. Grant M, Rees S, Underwood M, Froud R. Obstacles to returning to work with chronic pain: in-depth interviews with people who are off work due to chronic pain and employers. BMC musculoskeletal disorders. 2019;20(1):486.
22. Nortvedt L, Lohne V, Kumar BN, Hansen HP. A lonely life--A qualitative study of immigrant women on long-term sick leave in Norway. International journal of nursing studies. 2016;54:54-64.
23. Nortvedt L, Hansen HP, Kumar BN, Lohne V. Caught in suffering bodies: a qualitative study of immigrant women on long-term sick leave in Norway. Journal of clinical nursing. 2015;24(21-22):3266-75.
24. Brataas HV, Evensen AE. Life stories of people on sick leave from work because of mild mental illness, pain and fatigue. Work (Reading, Mass). 2015;53(2):285-91.
25. O'Brien BC, Harris IB, Beckman TJ, Reed DA, Cook DA. Standards for Reporting Qualitative Research: A Synthesis of Recommendations. 2014;89(9):1245-51.
26. Ricoeur P. Interpretation Theory: Discourse and the Surplus of Meaning: Texas Christian University Press; 1976.
27. Lindseth A, Norberg A. A phenomenological hermeneutical method for researching lived experience. Scandinavian Journal of Caring Sciences. 2004;18(2):145-53.
28. Alsén S, Ali L, Ekman I, Fors A. Facing a blind alley - Experiences of stress-related exhaustion: a qualitative study. BMJ open. 2020;10(9):e038230.
29. Wallström S, Ulin K, Omerovic E, Ekman I. Symptoms in patients with takotsubo syndrome: a qualitative interview study. BMJ open. 2016;6(10):e011820.
30. Söderberg S, Lundman B, Norberg A. Struggling for dignity: the meaning of women's experiences of living with fibromyalgia. Qualitative health research. 1999;9(5):575-87.
31. Polkinghorne DE. Phenomenological research methods. Existential-phenomenological perspectives in psychology: Exploring the breadth of human experience. New York, NY, US: Plenum Press; 1989. p. 41-60.
32. Association WM. World Medical Association Declaration of Helsinki: Ethical Principles for Medical Research Involving Human Subjects. JAMA. 2013;310(20):2191-4.
33. Ricœur P. Oneself as another. New ed. ed. Blamey K, editor: Chigago : University of Chicago Press; 1994.
34. Ekman I. Practising the ethics of person-centred care balancing ethical conviction and moral obligations. Nursing Philosophy. 2022:e12382.
35. van Rhenen W, Schaufeli WB, van Dijk FJ, Blonk RW. Coping and sickness absence. International archives of occupational and environmental health. 2008;81(4):461-72.
36. Svenaeus F. The body uncanny — Further steps towards a phenomenology of illness. Medicine, Health Care and Philosophy. 2000;3(2):125-37.
37. Paulson M, Danielson E, Söderberg S. Struggling for a Tolerable Existence: The Meaning of Men's Lived Experiences of Living with Pain of Fibromyalgia Type. 2002;12(2):238-49.

For peer review only - http://bmjopen.bmj.com/site/about/guidelines.xhtml

38. Geneen LJ, Martin DJ, Adams N, Clarke C, Dunbar M, Jones D, et al. Effects of education to facilitate knowledge about chronic pain for adults: a systematic review with meta-analysis. Systematic Reviews. 2015;4(1):132.
39. Lundberg M, Styf J, Bullington J. Experiences of moving with persistent pain--a qualitative study from a patient perspective. Physiotherapy theory and practice. 2007;23(4):199-209.
40. Merleau-Ponty M. Phenomenology of Perception: The Humanities Press; 1962.
41. De Ruddere L, Craig KD. Understanding stigma and chronic pain: a-state-of-the-art review. 2016;157(8):1607-10.
42. Smith BH, Fors EA, Korwisi B, Barke A, Cameron P, Colvin L, et al. The IASP classification of chronic pain for ICD-11: applicability in primary care. Pain (Amsterdam). 2019;160(1):83-7.
43. Wijnhoven HA, de Vet HC, Picavet HS. Prevalence of musculoskeletal disorders is systematically higher in women than in men. The Clinical journal of pain. 2006;22(8):717-24.
44. Sandelowski M. Sample size in qualitative research. Res Nurs Health. 1995;18(2):179-83.
45. Thunberg S, Arnell L. Pioneering the use of technologies in qualitative research – A research review of the use of digital interviews. International Journal of Social Research Methodology. 2021:1-12.

For peer review only

For peer review only - http://bmjopen.bmj.com/site/about/guidelines.xhtml

SAHLGRENSKA ACADEMY

Introduction

Presentation of the researchers, the project and its aim. Information about confidentiality, consent and withdrawal. Opportunity for the participants to ask questions.

Opening questions

- I understand you have been living in pain for a long time. Would you tell me about what this time has been like for you?
- In what way has being on sick leave and not being able to work affected your life?
- What would you say has been the biggest challenge for you as the person you think you are or want to be? Has being on sick leave changed the way you look at yourself?
- Are there things that could have prevented you from being on sick leave?

Work and support

- Having pain for a long time often affects the ability to work. In what way would you say that your pain has influenced your work situation?
- Everyone is different; some people want support to cope with their situation, and others feel they have the support they need to manage. How is it for you? What support did you need to deal with your situation?
- Do you feel that you have been given the opportunity for such support or adjustment at your workplace? If so, what has that support meant to you?

Self-efficacy and confidence in one's ability

- It is common to lose confidence in your abilities when you have been on sick leave for a long time. What do you think about that? Do you feel you can influence your situation yourself?
- Being on sick leave and living with pain can also affect how you feel about your body. Do you feel you can trust your body to do what you want to do? What are your thoughts about that?
- What do you think about the situation you are in today? How does your pain affect the goals and desires that you have in life?

Activity and participation

- Living with pain for a long time can often affect different aspects of everyday life. How do you feel it affects your leisure activities or social contacts?
- How has your pain affected your ability to sleep, rest or recover?

Conclusion

- Do you have any other thoughts or reflections about your situation that we have not discussed today and that you would like to add?

For peer review only

Reporting checklist for qualitative study.

Based on the SRQR guidelines.

Instructions to authors

Complete this checklist by entering the page numbers from your manuscript where readers will find each of the items listed below.

Your article may not currently address all the items on the checklist. Please modify your text to include the missing information. If you are certain that an item does not apply, please write "n/a" and provide a short explanation.

Upload your completed checklist as an extra file when you submit to a journal.

In your methods section, say that you used the SRQRreporting guidelines, and cite them as:

O'Brien BC, Harris IB, Beckman TJ, Reed DA, Cook DA. Standards for reporting qualitative research: a synthesis of recommendations. Acad Med. 2014;89(9):1245-1251.

		Reporting Item	Page Number
**Title**			
	#1	Concise description of the nature and topic of the study identifying the study as qualitative or indicating the approach (e.g. ethnography, grounded theory) or data collection methods (e.g. interview, focus group) is recommended	1
**Abstract**			
	#2	Summary of the key elements of the study using the abstract format of the intended publication; typically includes background, purpose, methods, results and conclusions	2
**Introduction**			
Problem formulation	#3	Description and signifcance of the problem / phenomenon studied: review of relevant theory and empirical work; problem statement	3-5

For peer review only

Purpose or research question	#4	Purpose of the study and specific objectives or questions	5
**Methods**			
Qualitative approach and research paradigm	#5	Qualitative approach (e.g. ethnography, grounded theory, case study, phenomenolgy, narrative research) and guiding theory if appropriate; identifying the research paradigm (e.g. postpositivist, constructivist / interpretivist) is also recommended; rationale. The rationale should briefly discuss the justification for choosing that theory, approach, method or technique rather than other options available; the assumptions and limitations implicit in those choices and how those choices influence study conclusions and transferability. As appropriate the rationale for several items might be discussed together.	5
Researcher characteristics and reflexivity	#6	Researchers' characteristics that may influence the research, including personal attributes, qualifications / experience, relationship with participants, assumptions and / or presuppositions; potential or actual interaction between researchers' characteristics and the research questions, approach, methods, results and / or transferability	7
Context	#7	Setting / site and salient contextual factors; rationale	5
Sampling strategy	#8	How and why research participants, documents, or events were selected; criteria for deciding when no further sampling was necessary (e.g. sampling saturation); rationale	5-6, 19
Ethical issues pertaining to human subjects	#9	Documentation of approval by an appropriate ethics review board and participant consent, or explanation for lack thereof; other confidentiality and data security issues	7
Data collection methods	#10	Types of data collected; details of data collection procedures including (as appropriate) start and stop dates of data collection and analysis, iterative process, triangulation of sources / methods, and modification of procedures in response to evolving study findings; rationale	6

For peer review only

Data collection instruments and technologies	#11	Description of instruments (e.g. interview guides, questionnaires) and devices (e.g. audio recorders) used for data collection; if / how the instruments(s) changed over the course of the study	6
Units of study	#12	Number and relevant characteristics of participants, documents, or events included in the study; level of participation (could be reported in results)	7-8
Data processing	#13	Methods for processing data prior to and during analysis, including transcription, data entry, data management and security, verification of data integrity, data coding, and anonymisation / deidentification of excerpts	6-7
Data analysis	#14	Process by which inferences, themes, etc. were identified and developed, including the researchers involved in data analysis; usually references a specific paradigm or approach; rationale	6-7
Techniques to enhance trustworthiness	#15	Techniques to enhance trustworthiness and credibility of data analysis (e.g. member checking, audit trail, triangulation); rationale	6-7
**Results/findings**			
Syntheses and interpretation	#16	Main findings (e.g. interpretations, inferences, and themes); might include development of a theory or model, or integration with prior research or theory	7-15
Links to empirical data	#17	Evidence (e.g. quotes, field notes, text excerpts, photographs) to substantiate analytic findings	7-14
**Discussion**			
Intergration with prior work, implications, transferability and contribution(s) to the field	#18	Short summary of main findings; explanation of how findings and conclusions connect to, support, elaborate on, or challenge conclusions of earlier scholarship; discussion of scope of application / generalizability; identification of unique contributions(s) to scholarship in a discipline or field	15-20
Limitations	#19	Trustworthiness and limitations of findings	19-20

For peer review only

Other

Conflicts of interest	#20	Potential sources of influence of perceived influence on study conduct and conclusions; how these were managed	21
Funding	#21	Sources of funding and other support; role of funders in data collection, interpretation and reporting	21

The SRQR checklist is distributed with permission of Wolters Kluwer © 2014 by the Association of American Medical Colleges. This checklist was completed on 14. June 2019 using https://www.goodreports.org/, a tool made by the EQUATOR Network in collaboration with Penelope.ai

For peer review only

BMJ Open

Suffering out of sight but not out of mind—interpreting experiences of sick leave due to chronic pain in a community setting: a qualitative study

Journal:	*BMJ Open*
Manuscript ID	bmjopen-2022-066617.R2
Article Type:	Original research
Date Submitted by the Author:	28-Mar-2023
Complete List of Authors:	Lundin, Åse; University of Gothenburg Institute of Health and Care Sciences, Sahlgrenska Academy, University of Gothenburg; University of Gothenburg Centre for Person-Centred Care, Sahlgrenska Academy, University of Gothenburg<br>Ekman, Inger; University of Gothenburg Institute of Health and Care Sciences, Sahlgrenska Academy, University of Gothenburg; University of Gothenburg Centre for Person-Centred Care, Sahlgrenska Academy, University of Gothenburg<br>Wallström, Sara; University of Gothenburg Institute of Health and Care Sciences, Sahlgrenska Academy, University of Gothenburg; University of Gothenburg Centre for Person-Centred Care, Sahlgrenska Academy, University of Gothenburg<br>Andréll, Paulin; Sahlgrenska University Hospital Ostra Hospital, Department of Anaesthesiology and Intensive Care Medicine/Pain Centre; University of Gothenburg Institute of Clinical Sciences, Department of Anaesthesiology and Intensive Care Medicine, Sahlgrenska Academy<br>Lundberg, Mari; Sophiahemmet University, Department of Health Promoting Science; University of Gothenburg Centre for Person-Centred Care, Sahlgrenska Academy, University of Gothenburg
**Primary Subject Heading**:	Rehabilitation medicine
Secondary Subject Heading:	Qualitative research, Public health
Keywords:	QUALITATIVE RESEARCH, PAIN MANAGEMENT, PUBLIC HEALTH, REHABILITATION MEDICINE

SCHOLARONE™
Manuscripts

I, the Submitting Author has the right to grant and does grant on behalf of all authors of the Work (as defined in the below author licence), an exclusive licence and/or a non-exclusive licence for contributions from authors who are: i) UK Crown employees; ii) where BMJ has agreed a CC-BY licence shall apply, and/or iii) in accordance with the terms applicable for US Federal Government officers or employees acting as part of their official duties; on a worldwide, perpetual, irrevocable, royalty-free basis to BMJ Publishing Group Ltd ("BMJ") its licensees and where the relevant Journal is co-owned by BMJ to the co-owners of the Journal, to publish the Work in this journal and any other BMJ products and to exploit all rights, as set out in our licence.

The Submitting Author accepts and understands that any supply made under these terms is made by BMJ to the Submitting Author unless you are acting as an employee on behalf of your employer or a postgraduate student of an affiliated institution which is paying any applicable article publishing charge ("APC") for Open Access articles. Where the Submitting Author wishes to make the Work available on an Open Access basis (and intends to pay the relevant APC), the terms of reuse of such Open Access shall be governed by a Creative Commons licence – details of these licences and which Creative Commons licence will apply to this Work are set out in our licence referred to above.

Other than as permitted in any relevant BMJ Author's Self Archiving Policies, I confirm this Work has not been accepted for publication elsewhere, is not being considered for publication elsewhere and does not duplicate material already published. I confirm all authors consent to publication of this Work and authorise the granting of this licence.

For peer review only

Suffering out of sight but not out of mind—interpreting experiences of sick leave due to chronic pain in a community setting: a qualitative study

Åse Lundin (ORCID iD 0000-0001-9980-7653)

- Institute of Health and Care Sciences, Sahlgrenska Academy, University of Gothenburg, Gothenburg, Sweden
- University of Gothenburg Centre for Person-Centred Care (GPCC), Sahlgrenska Academy, University of Gothenburg, Gothenburg, Sweden

Inger Ekman (ORCID iD 0000-0002-5559-5203)

- Institute of Health and Care Sciences, Sahlgrenska Academy, University of Gothenburg, Gothenburg, Sweden
- University of Gothenburg Centre for Person-Centred Care (GPCC), Sahlgrenska Academy, University of Gothenburg, Gothenburg, Sweden

Sara Wallström (ORCID iD 0000-0001-7579-4974)

- Institute of Health and Care Sciences, Sahlgrenska Academy, University of Gothenburg, Gothenburg, Sweden
- University of Gothenburg Centre for Person-Centred Care (GPCC), Sahlgrenska Academy, University of Gothenburg, Gothenburg, Sweden
- Forensic psychiatric department, Sahlgrenska University Hospital, Gothenburg, Sweden

Paulin Andréll (ORCID iD 0000-0001-6941-6096)

- Region Västra Götaland, Sahlgrenska University Hospital/Östra, Department of Anaesthesiology and Intensive Care Medicine/Pain Centre, Gothenburg, Sweden
- Department of Anaesthesiology and Intensive Care Medicine, Institute of Clinical Sciences at Sahlgrenska Academy, University of Gothenburg, Gothenburg, Sweden

Mari Lundberg (ORCID iD 0000-0001-7123-0362)

- Department of Health Promoting Science, Sophiahemmet University, Stockholm, Sweden
- University of Gothenburg Centre for Person-Centred Care (GPCC), Sahlgrenska Academy, University of Gothenburg, Gothenburg, Sweden

Correspondence to:

Åse Lundin

Arvid Wallgrens backe, hus 1, Box 457, 405 30 Göteborg, Sweden

For peer review only - http://bmjopen.bmj.com/site/about/guidelines.xhtml

ase.lundin@gu.se

Word count, excluding title page, abstract, declarations, references and tables: 4911

Abstract

Objective: Chronic pain is a complex health problem affecting about one fifth of the European population. It is a leading cause of years lived with disability worldwide, with serious personal, relational, and socio-economic consequences. Chronic pain and sick leave adversely affect health and quality of life. Thus, understanding this phenomenon is essential for reducing suffering, understanding the need for support, and promoting a rapid return to work and an active lifestyle. This study aimed to describe and interpret persons' experiences of being on sick leave due to chronic pain.

Design: A qualitative study with semi-structured interviews analysed using a phenomenological hermeneutic approach.

Setting: Participants were recruited from a community setting in Sweden.

Participants: Fourteen participants (12 women) with experiences of part- or full-time sick leave from work due to chronic pain were included in the study.

Findings: *Suffering out of sight but not out of mind* was the main theme of the qualitative analysis. This theme implies that the participants' constant suffering was invisible to others, causing them to feel they were not being justly treated in society. Feeling overlooked led to a continuous struggle for recognition. Moreover, the participants' identities and their trust in themselves and their bodies was challenged. However, our study also revealed a nuanced

For peer review only - http://bmjopen.bmj.com/site/about/guidelines.xhtml

understanding of the experiences of sick leave as a consequence of chronic pain, where the participants learned important lessons, including coping strategies and re-evaluated priorities.

Conclusions: Being on sick leave due to chronic pain threatens a person's integrity and leads to substantial suffering. An enhanced understanding of the meaning of sick leave due to chronic pain provides important considerations for their care and support. This study highlights the importance of feeling acknowledged and being met with justice in encounters with others.

Keywords:

Chronic pain, sick leave, qualitative research, phenomenological hermeneutics, lived experiences

For peer review only

Strengths and limitations of this study

- Qualitative research is important in enlightening the personal experiences of pain and its consequences, uncovering information not accessible through other methods.
- Exploring the meanings of lived experiences can help us understand and improve our practices in health care.
- Studies using interpretative qualitative methods are encouraged within the field of chronic pain and address a knowledge gap in the existing literature.
- In this study only two out of the 14 participants were men, yielding a distribution that might have affected our findings.

Introduction

For peer review only - http://bmjopen.bmj.com/site/about/guidelines.xhtml

Chronic pain has reached alarming levels, particularly among older adults, and is one of the leading causes of years lived with disability (1). Pain of moderate-to-severe intensity affects 19% of the European population (2). The socio-economic burden of chronic pain is substantial, mainly for working-age people, with the bulk of indirect costs related to absence from the workforce (3). In the US the total cost associated with chronic pain in adults ranges from 560 to 635 billion USD, exceeding the annual costs estimated for heart disease (309 billion USD), cancer (243 billion USD), and diabetes (188 billion USD) (4). In Sweden, the cost of chronic pain diagnoses was estimated to be 32 billion euros annually (3). Chronic pain has a significant impact on quality of life and is closely associated with depression (5), increased suicide rates (6), premature mortality and reduced life expectancy (7, 8). Because a key feature of pain is that it is a "*personal experience that is influenced to varying degrees by biological, psychological, and social factors"* (9), a person's experience or report of pain is of particular interest and should be taken seriously (10). Qualitative pain research has been increasing in recent years, exposing aspects of pain experience that is not accessible through other approaches (11). Previous studies of people's experiences with chronic pain revealed feelings of restriction, loss and limitation of daily life (12, 13), loneliness, and silent suffering (14). These studies also showed that chronic pain had a pronounced effect on relationships, family life, and work (13). In addition, being on sick leave negatively affects a person's life. Research on the experiences of work absenteeism because of illness has been done in different populations and for different health conditions, revealing negative experiences including loss of identity (15), social exclusion, and a struggle to adapt to reduced work capacity (16). Being on sick leave also led to feelings of uncertainty and insecurity (17), stigma, helplessness, and a loss of independence (18).

Despite implementing evidence-based practices (e.g., interdisciplinary rehabilitation), many persons with chronic pain do not return to work (19). Qualitative pain research has often

For peer review only - http://bmjopen.bmj.com/site/about/guidelines.xhtml

focused on the barriers and facilitators people with chronic pain face when returning to work (20, 21). However, there is limited research on the personal experiences of being on sick leave due to chronic pain. Some studies reflect on immigrant women's experiences of long-term sick leave due to chronic pain. These studies found that the women felt excluded and rejected (22), struggling with an invisible physical ailment (23). Other studies have explored the phenomenon on a descriptive level (24). Yet, researchers have been encouraged to go beyond description and towards interpretative engagement while performing qualitative analyses of pain (11). In line with this approach, we sought to understand the essential meaning of sick leave due to chronic pain. A deepened understanding of the personal experiences of the phenomenon is vital to reducing suffering, better understanding their needs, and facilitating a return to working life.

This study aimed to describe and interpret persons' experiences of sick leave due to chronic pain.

Methods

The authors followed the Standards for Reporting Qualitative Research guidelines (25) to ensure the trustworthiness of the findings.

Design

In this study we chose a qualitative interview design to explore participants' experiences of living with chronic pain and on sick leave. Data (transcripts of semi-structured interviews) were analysed using phenomenological hermeneutics inspired by Ricoeur (26) and developed by Lindseth and Norberg (27). The method has been used in numerous health and care studies

For peer review only - http://bmjopen.bmj.com/site/about/guidelines.xhtml

(28-30). The method aims to understand the meaning of the participants' lived experiences by interpreting their narratives.

Participants and setting

The inclusion criteria for this study were: having experience of part- or full-time sick leave attributable to chronic pain (duration of pain >3 months), speaking Swedish or English and being able and willing to participate in an interview. The recommended target size in qualitative interview studies varies, but phenomenological research often involves 5 to 25 participants who have experienced the phenomenon in question (31). During the interviews, three of the present authors (ÅL, SW and IE) regularly evaluated the richness of the collected data relating to the research aim to help determine when sufficient participants had been included.

The participants were recruited through patient organisations and associations focusing on pain or pain-related conditions. A request for participation was sent through their e-mail, newsletter, or Facebook pages. The participants could choose the interview location and whether they wanted to meet face-to-face or through digital communication.

Data collection

Data were collected via individual semi-structured in-depth interviews that were audio-recorded and transcribed verbatim. All interviews and transcripts were confidential using numerical coding, and precautions were taken to safeguard the identity of the participants.

An interview guide was used (see supplemental material). All interviews opened with the question, *"I understand you have been living in pain for a long time. Would you tell me what this time has been like for you?."* Follow-up and probing questions such as "*Can you give me an example?"* and *"How did that make you feel?"* encouraged further narration. The interview guide was not used as a strict list of questions, but to help propel the conversation

For peer review only - http://bmjopen.bmj.com/site/about/guidelines.xhtml

For peer review only

forward and ensure the research questions were covered during the interview. Some participants freely narrated, while others needed more guidance to address the research questions.

Analysis and interpretation of data

Phenomenological hermeneutics comprises three interrelated parts: naïve reading, structural analysis, and interpretation of the whole (27). During the naïve reading, the text was read repeatedly to grasp its meaning as a whole. In the structural analysis the text related to the aim of the study was divided into meaning units that were then abstracted and formed into subthemes, themes, and a main theme. Qualitative data analysis software (QSR NVivo R1) was used in the structural analysis. The analysis was a continuous process moving between the parts and meaning units of the text and the overall understanding. Three authors (ÅL, ML, and IE) met regularly during this process to discuss the collected data and the emergence of subthemes, themes, and the main theme to highlight different perspectives, add breadth and nuances, and ensure a congruent reflection and understanding of the narratives. All parts of the text relevant to the research question were included in the analysis. During the final part of the analysis, the text was reread. The initial naïve reading, the structural analysis and the authors' preunderstandings were combined to interpret the overall meaning of the text. We used research related to the area, theoretical literature, and discussions among the authors to gain a better understanding of the text.

Research characteristics and reflexivity

ÅL is a physiotherapist with several years of clinical experience in primary health care. The author has frequently encountered patients seeking care for chronic pain who cannot work because of their condition. ÅL has previously done qualitative research but not in areas related to pain. Of the co-authors, two have extensive experience in research on pain (ML and

For peer review only - http://bmjopen.bmj.com/site/about/guidelines.xhtml

PA) and three have extensive experience working with qualitative analyses (IE, ML, and SW).

Ethical considerations

Written consent was obtained from each participant. The study was approved by the Swedish Ethical Review Authority DNR 2020-02491 and complied with the principles in the Declaration of Helsinki (32).

Patient and public involvement

Before the study, several patient associations were contacted to help recruit participants. These patient organisations and their channels will also disseminate the study results to reach a wider audience beyond the academic community.

Findings

Of the 18 people who contacted the first author (ÅL) regarding participation, 3 decided not to go through with an interview and 1 did not attend the booked interview. Fourteen participants (12 women) were included in the study and were interviewed. Participant characteristics are listed in Table 1.

Table 1. Participant characteristics

**Characteristics**	**(n = 14)**
Men/Women	2/12
Mean age, years (range)	49 (23-80)
Current work status	

For peer review only

Full-time sick leave	4
Part-time sick leave	5
Returned to work	4
Retired	1
Highest completed education	
Primary school (9 years)	2
High School	3
Other upper secondary education	4
University degree	5
Duration of pain	
1-3 years	2
4-9 years	1
>10 years	11

The location and causes of chronic pain varied among the participants. Low back pain, neck pain, rheumatism, fibromyalgia, shoulder pain, arthritis, endometriosis, joint pain due to Ehlers-Danlos syndrome, whiplash injury, and spinal injury were represented among the participants.

The interviews, lasting 41-90 minutes, were conducted in 2021 by the first author. Of the 14 interviews, 10 were performed through digital channels, 2 in the participants' homes, and 2 on university premises.

Naïve reading

The naïve reading of the text indicated that living with chronic pain and being on sick leave significantly impacted the participants' lives and how they identified themselves. The feelings of exclusion, loss, and unfamiliarity prevailed throughout the naïve understanding. The

For peer review only - http://bmjopen.bmj.com/site/about/guidelines.xhtml

participants struggled to manage a condition experienced as invisible to others, hindering participation in daily activities and work participation.

Structural analysis

The structural analyses partly validated our first impressions. These analyses also helped nuance and emphasise other aspects of the phenomenon, mainly regarding dependency and relations with institutions, understandings of the phenomenon that went unnoticed in the first part of the analysis.

An overview of the main theme, themes and subthemes from the structural analysis is given in Table 2 and further described below.

Table 2. Overview of the main theme, themes and subthemes

**Suffering out of sight but not out of mind**		
**An identity under threat**	**Trapped in a foreign body**	**Navigating uncharted waters**
Becoming dependent<br>Being excluded<br>Feeling flawed	Losing bodily trust<br>Feeling constantly tired	Constantly chasing recognition<br>A journey towards unexpected lessons

An identity under threat

Being on sick leave because of chronic pain affected and threatened the participants' views of themselves and their identity, significantly influencing their relationships with others. This theme was expressed in three subthemes: Becoming dependent, Being excluded, and Feeling flawed.

For peer review only - http://bmjopen.bmj.com/site/about/guidelines.xhtml

1. Becoming dependent

The participants felt they no longer recognise themselves concerning their capabilities. They had previously viewed themselves as independent and able to care for and fend for themselves. However, being on sick leave due to chronic pain made them increasingly dependent on others. Becoming dependent involves relying on help from family and friends, outsourcing physical household chores, and becoming economically reliant on their partners or others to pay for their dwelling or provide for the children. The participants experienced frustration from relying on the benefits and goodwill of authorities concerning their work status and sick leave payments. Decisions were often out of the participants' control, causing distress and feelings of worthlessness and powerlessness. One participant who was denied access to sick leave benefits said,

Ultimately, you feel like you are just a burden to your husband [crying]. I have no income to get by myself. I cannot earn a living. And if he cannot provide service, help me, or fix the car, we have to sell it because I can't do it. # 4

2. Being excluded

The presence of pain and being on sick leave led to feelings of exclusion from the workplace and losing value as an employee and person. The participants had previously taken pride in being willing to work hard. They wanted to return to work and be a part of the social life and the routines they had at the workplace that made them feel meaningful and appreciated. Having to go on sick leave was experienced as imprisonment or punishment and feeling left behind.

Being at home it…I think it is the worst punishment. The days…the days are long when you are healthy so you can imagine when you are sick and restricted

For peer review only - http://bmjopen.bmj.com/site/about/guidelines.xhtml

and…and being alone in the house and my husband is working and my daughter is at school and then you think a lot of negative thoughts and you have to occupy the brain, I guess. #14

3. Feeling flawed

Because the participants lived with pain and could not work, they felt flawed as persons, parents, and participants in daily life. They struggled with a loss of engagement, unhappiness, increased anger, a short temper towards family members, and other personality changes. They often had to decline participation in social activities or hobbies against their wishes. It affected their relationships with family and friends, creating grief, largely concerning being unable to be there for their friends, spouse, or children as they used to be.

But you still feel that it's like you are not sufficiently there. So, you also have a constant feeling of guilt that "no, mommy isn't able to…". #6

Trapped in a foreign body

Suffering from chronic pain meant not recognising the body as the familiar place it used to be, creating conflicts that made it difficult to cope with daily and working life. This theme was expressed in two subthemes: Losing bodily trust and Feeling constantly tired.

1. Losing bodily trust

Experiencing distressing physical symptoms combined with intensive and unreliable pain fluctuations was tiresome for the participants. Not knowing how their body would function or react daily created a loss of trust and a feeling that their body was not the safe and trusted

For peer review only - http://bmjopen.bmj.com/site/about/guidelines.xhtml

home it once was. One participant described it as always wearing a heavy diving suit, interpreted as a conflict between what the participant wanted and what the body could do. Another participant who worked as a caretaker, described how the rapid increase in pain intensity made her feel insecure and lose trust in her body while helping and interacting with the elderly in their homes.

Then I would make supper and help them into their nightclothes, and then you ought to…some of them who were in wheelchairs would have toilet visits and then do many things. And I dropped pots and cups on sinks, and they made a bang and these older people, 80-90 years old, were terrified! And I got so much pain that I could barely lift this pot back up again, or the cup or… I mean, I would have hot, both hot water and stuff. I'm terrified that I would drop an old…/… /And then I did not want to go back there when I know I do not, that I cannot hold. How can I hold a whole person when I cannot even grab a cup? # 4

2. Feeling constantly tired

The participants experienced that fatigue and the inability to sleep were a greater disturbance in their daily lives and their ability to work than the pain itself. The little energy possibly gained through the night was quickly drained the next day when coping with the fatigue associated with the pain. Sleepless nights, the side effects of sleep medication, and not feeling rested were associated with stress and suffering. Being on sick leave and not having to get up for work opened up the possibility of resting a little during the day instead of the night, which was interpreted as a small window of opportunity to find relief.

Because it's, it's, it's actually the worst, really. The pain, you can do something about it, you can, it can be eased...but the fatigue cannot be relieved in the same way. #10

For peer review only - http://bmjopen.bmj.com/site/about/guidelines.xhtml

Navigating uncharted waters

Living in pain and not being able to participate in work was interpreted as navigating through the unfamiliar, a route filled with unpleasantness and uncertainties (but sometimes helpful surprises). This theme was expressed in two subthemes: Constantly chasing recognition and A journey towards unexpected lessons.

1. Constantly chasing recognition

Living with pain meant confronting mistrust or suspicions from different parts of society. The pain was very tangible for the participants, but they experienced their condition as hidden from the outside world. This perceived invisibility made the participants feel invariably questioned, especially by the authorities, the healthcare system, and the workplace. They felt that they were seen as complainers, lazy, and making up or exaggerating their symptoms, which led to an endless pursuit to be believed and have their suffering recognised and taken seriously. One participant described not being met with support from the health care system early enough and how that might have prolonged her sick leave.

> *If the scenario had been such that the healthcare system took care of me and helped me, I wouldn't have had... even though I will always have problems because I'm chronically ill, I will always have problems, but it hadn't been that noticeable. It would not have been so extensive and so often and so strong, which would have meant that I could have been able to handle it and I would have been able to work # 9*

Instead, the participants continuously fought to receive a diagnosis that could be shown through objective assessment to explain their pain and validate their getting access to treatment and their leave of absence from work.

For peer review only - http://bmjopen.bmj.com/site/about/guidelines.xhtml

*It does not matter what I say because it feels like they see my smile and that I am active and walking that "well, but then she is not so ill." And if I had gone back to work, "but then you are not so ill," and it starts all over again!/…./Just because it is not visible on a damn test…That you couldn't just, you know, you can connect cars to computers, couldn't you get a frigging body scan and *swoosh* where it lights up red, she's in pain there! # 3*

For peer review only

2. A journey towards unexpected lessons

The participants were forced to find different coping strategies and new routines because of the unrelenting fatigue and constant pain. This endeavour was interpreted as a bumpy journey with uncertainties and worries, especially regarding their future and work situation if their condition deteriorates. The participants' experiences of living with pain were not all undesirable, as some had learned meaningful lessons along the way. They gained new insights into the importance of not pushing oneself too hard, that rest is essential, and that it is not beneficial to let work dominate life. They identified abilities and resources within themselves that they had not previously noticed that helped them cope with their new situation. They also re-evaluated their priorities regarding relationships and family and the importance of appreciating the small things in life.

I think I'm better now at thinking that the small things are good and pleasant. It may be enough that I go for a walk in the woods and look at the water. You will still be all right, or better. In a way that I didn't …I didn't see the qualities of the little things in life quite the same way. Maybe it was the bigger things that were more important. The rest was mostly just everyday things. I do not think that way

For peer review only - http://bmjopen.bmj.com/site/about/guidelines.xhtml

anymore; instead, I believe that there are small things that are important after all. #8

Interpretation of the whole

The naïve reading, the structural analysis, and the authors' pre-understandings were integrated to interpret the overall meaning of the text material. The interpretation was inspired by Ricoeur's philosophy and ethical intentions of *"aiming at a good life with and for others in just institutions"* (33), emphasising that ethics is prioritised over morality or norms. Ricoeur's philosophy on just institutions is of particular interest to the current study as it is essential that people feel justly treated in their contact with institutions (34) such as the healthcare system, the authorities, or the workplace. With pain often experienced as an invisible illness to others, there was a continual pursuit to have their life situation validated and recognized. We interpret this as being about justice, a feeling that they were not receiving or being met with the justice they deserved. During their sick leave, the participants' identities were threatened by feelings of flaws, exclusion, and dependency. They felt trapped in their bodies, which they experienced as foreign and thus not their own. The themes were interpreted as being connected by processes invisible to others, including institutions, but still brought much suffering to the participants. This suffering was exacerbated by the feelings of not being treated fairly. Although the participants' struggles were hidden from the outside world, they were real to the participants, affecting all aspects of life. Based on this, the meaning of living with and being on sick leave due to chronic pain was interpreted as *Suffering out of sight but not out of mind,* which is the main theme. It is a play on the idiom "out of sight, out of mind," meaning that you soon forget people (or things) that are no longer

For peer review only - http://bmjopen.bmj.com/site/about/guidelines.xhtml

visible or present. For the participants, however, the pain and suffering were permanent and real.

Discussion

The main finding of this study is that the participants experienced their suffering as invisible to others, causing them to feel that they were not being justly treated in society and invariably fighting for recognition of their condition. Moreover, being on sick leave due to chronic pain challenged the participants' identities, leading to a loss of trust in themselves and their bodies. Contrary to previous research, which has mainly underlined the hardships and adversities of sick leave (16, 17, 22, 23), our study revealed a nuanced understanding of the experiences of sick leave due to chronic pain. Despite their difficulties, and sometimes to their surprise, the participants felt that this situation had taught them new things about themselves, forced them to develop new coping-strategies, and allowed them to embrace other aspects of life. Research has shown that supportive experiences (e.g., meeting respectful health care professionals) are important to those on sick leave (18) and that possessing problem-solving coping strategies significantly reduces sick leave (35). In our study we also interpreted the participants' learning experiences as something that stretched over a broader context and that adds value to relationships, family life, and a positive approach to life. Our findings are important because if the participants had gained these skills and coping strategies earlier, their sick leave may possibly have been shortened or prevented. The participants in our study also explained that they might have continued working if their struggles had been recognised at the workplace or by the healthcare system from the start. We interpret this inadequacy as lacking the institutional support the participants needed.

For peer review only - http://bmjopen.bmj.com/site/about/guidelines.xhtml

The experience of being in a foreign body when suffering from poor health has been described in other studies. In illness the body can be perceived as ‘unhomelike’ and as a ‘broken tool’ no longer in control, resulting in a loss of command over one's own body (36). Suffering from chronic pain could also be interpreted as experiencing the body as an obstruction and being confronted with having a body that feels different from when healthy (37). Our participants noted how they were affected by several neurophysiological changes due to pain, including fatigue, stress, and mood changes (e.g., increased anger). According to our clinical experiences this might represent a lack of knowledge of the dialectical relationship between the body and psychological or behavioural changes. Increasing the understanding of the relationship between the mind and body and how this interaction affects the pain experience is an integral part of pain rehabilitation. Pain neuroscience education has been shown to increase this knowledge and reduce disability among patients suffering from chronic pain (38). Our study also found that the losing familiarity with the body when suffering from pain hindered the participants' return to work. The ability to return to work was not only a question of the physical strength needed to perform certain work tasks, but about trusting their capabilities and feeling safe in their bodies in the workplace setting. As shown in previous research, the experience of moving while in persistent pain implies more than just physical restriction. It challenges and threatens a person's lifeworld and existence (39). In the words of Merleau-Ponty, our lived body is our access to or *"a vehicle of being in the world"* (40). When our body changes (i.e., when trapped in an unknown body due to pain) it alters how the world is perceived.

Our participants viewed their identities as challenged or under threat while on sick leave owing to chronic pain. This feeling is consistent with previous findings on the sick leave process due to other health conditions, expressed as being reduced as a person and not recognising oneself (15) or experiencing a changed self-perception (18). Our participants

For peer review only - http://bmjopen.bmj.com/site/about/guidelines.xhtml

depicted a strong connection between identity and work. Losing contact with the workplace meant forfeiting who they were as productive elements of society. The authenticity of pain conditions as a cause of work absence has been questioned by some employers, suggesting that workers are ‘lazy’ (21). In contrast, our study shows that the participants had a strong desire to return to work and experience the benefits that were linked to being part of the workforce.

We interpreted the experiences of constantly chasing recognition while facing distrust and suspicion from others as not being met by just institutions. A just institution means that each person, when in contact with, for example, the healthcare system senses justice on a fundamental level, i.e., is treated well and considered an equal partner in the care process (34). A just institution, a crucial part of person-centred care, allows persons to express their needs and abilities and can even offer a sense of partnership (34). However, our participants reported feeling subservient and helpless, with their lives dependent on the goodwill of institutional gatekeepers. Previous research on sick leave has observed similar experiences, i.e., instead of a partnership, the rules and regulations make the stakeholders become counterparts, leaving little flexibility for the person (15). But being on sick leave due to chronic pain was also interpreted as different from other health issues, mainly because of its invisibility and the lack of objective assessment of pain symptoms. A social stigma is attached to chronic pain (41). In our study, one participant wondered whether the workplace or authorities would have looked at her differently if she had cancer rather than a chronic pain condition. In the 2018 International Classification of Diseases 11th revision (ICD-11) a coding system for chronic pain was included for the first time (42). Acknowledging and recognising chronic pain as a disease in itself and not just a symptom is an opportunity to improve treatment and our understanding of chronic pain and its management (42). Another benefit is that this new classification may potentially shift the focus from finding a cause to

For peer review only - http://bmjopen.bmj.com/site/about/guidelines.xhtml

managing the impact of the chronic pain for the patient (e.g., through multimodal treatments) (42). The participants in this study often fought hard to receive a diagnosis and to be able to objectively prove that they had pain to the health care professionals in order to receive the right help. The new ICD-11 can hopefully be part of acknowledging the importance of understanding in rehabilitation and in the process of returning to work, as well as potentially reducing stigma by increasing the legitimacy of chronic pain.

Methodological considerations

In the current study only 2 of the 14 participants were men. This skewed distribution might partly be explained by research showing that women report a higher prevalence of chronic pain (43) but could also be influenced by other factors (e.g., women being overrepresented or more active in the patient organisations used to recruit participants). The study also explored the 14 participants' narratives, and their experiences might not apply to the experiences of other persons on sick leave as a result of chronic pain. However, qualitative research does not argue that the findings are universal and generalisable; rather, it explores possible meanings of lived experiences to understand and improve our practices in health care (27).

Sample size in qualitative studies is often a subject of discussion, where no power calculations can determine the sample size needed (44). In phenomenological research sample sizes of between 5 and 25 persons are commonly suggested (31). But determining sample size and when no further sampling is needed is a matter of judgment and experience in evaluating the quality of already collected data to the aims and methods used (44). During the inclusion process and inspection of the interview data, the material was discussed within the research group and deemed to contain rich and varied narratives of the participants' experiences. Thus, the interview data sufficed to answer the research question using the

For peer review only

For peer review only - http://bmjopen.bmj.com/site/about/guidelines.xhtml

phenomenological hermeneutic method. Given the richness of the interview data, further analyses that focus on other aspects of the collected data are planned.

Another potential limitation is related to the COVID-19 pandemic restrictions. Because of the restrictions, most interviews were performed digitally, which could have resulted in differences in the interactions and dynamics during the interviews compared to face-to-face encounters. A recent review showed that digital interviews could adversely affect the interviews, including experiencing technical issues or difficulties reading visual cues or body language. Yet, the review also found that richer data could be extracted through a digital approach compared to face-to-face interviews, when investigating sensitive topics (e.g., health issues), most likely because the participants felt more in control of the situation and the setting and thus felt more relaxed (45).

Conclusions

This study underscores the experiences of persons on sick leave due to chronic pain, where feelings of being invisible and overlooked often dominate. They do not feel that they are met with justice in their encounters with their surroundings which creates existential concerns and threatens their identities, bodies, and interactions with others. In contrast to previous research, which has mainly spotlighted the adversities of being in the sick leave process, our study revealed a nuanced understanding of the participants' experiences of sick leave due to chronic pain. A deepened understanding of this phenomenon provides important considerations for the care and support of these persons, as well as means for joint planning of rehabilitation towards a return to work. It is important to understand their lifeworld to acknowledge and raise the issues of existential threat, stigma, invisibility, and feelings of injustice that these persons experience in encounters with others. Suggestions for future research include further investigating what factors might facilitate a return to work after

For peer review only - http://bmjopen.bmj.com/site/about/guidelines.xhtml

being on sick leave due to chronic pain and exploring the impact of the new ICD-11 classification on the personal experiences of living with chronic pain.

Declarations

- **Ethics approval**

  The study was approved by the Swedish Ethical Review Authority DNR 2020-02491 and conformed to the Declaration of Helsinki (32). At the start of the study, written informed consent was obtained from each participant.

- **Funding**

  This work was supported by FORTE grant number 2019-00718 and the University of Gothenburg Centre for Person-Centred Care (GPCC) grant number N/A.

- **Contributors**

  All authors participated in the conception and design of the study. ÅL recruited participants, conducted and transcribed the interviews, and performed the analysis and interpretation of data with critical input from IE and ML. The manuscript was drafted by ÅL and critically revised and edited by IE, ML, SW, and PA. All authors read and approved the final version of the manuscript and agree to be accountable for all aspects of the work in ensuring that questions related to the accuracy or integrity of any part of the work are appropriately investigated and resolved.

- **Competing interests**

  None declared.

- **Data availability statement**

  No additional data available.

- **Patient consent for publication**

For peer review only - http://bmjopen.bmj.com/site/about/guidelines.xhtml

Not required.

- **Acknowledgements**

We acknowledge the participants for taking the time and effort to share their narratives and experiences.

References

1. Rice ASC, Smith BH, Blyth FM. Pain and the global burden of disease. PAIN. 2016;157(4).
2. Breivik H, Collett B, Ventafridda V, Cohen R, Gallacher D. Survey of chronic pain in Europe: prevalence, impact on daily life, and treatment. European journal of pain (London, England). 2006;10(4):287-333.
3. Gustavsson A, Bjorkman J, Ljungcrantz C, Rhodin A, Rivano-Fischer M, Sjolund K-F, et al. Socio-economic burden of patients with a diagnosis related to chronic pain – Register data of 840,000 Swedish patients. 2012;16(2):289-99.
4. Gaskin DJ, Richard P. The economic costs of pain in the United States. The journal of pain. 2012;13(8):715-24.
5. Bair MJ, Robinson RL, Katon W, Kroenke K. Depression and Pain Comorbidity: A Literature Review. Archives of Internal Medicine. 2003;163(20):2433-45.
6. Tang NKY, Crane C. Suicidality in chronic pain: a review of the prevalence, risk factors and psychological links. Psychological Medicine. 2006;36(5):575-86.
7. Torrance N, Elliott AM, Lee AJ, Smith BH. Severe chronic pain is associated with increased 10 year mortality. A cohort record linkage study. 2010;14(4):380-6.
8. Smith D, Wilkie R, Uthman O, Jordan JL, McBeth J. Chronic Pain and Mortality: A Systematic Review. PLOS ONE. 2014;9(6):e99048.
9. Raja SN, Carr DB, Cohen M, Finnerup NB, Flor H, Gibson S, et al. The revised International Association for the Study of Pain definition of pain: concepts, challenges, and compromises. 2020;161(9):1976-82.
10. Cohen SP, Vase L, Hooten WM. Chronic pain: an update on burden, best practices, and new advances. The Lancet. 2021;397(10289):2082-97.
11. Osborn M, Rodham K. Insights into Pain: A Review of Qualitative Research. Rev Pain. 2010;4(1):2-7.

For peer review only - http://bmjopen.bmj.com/site/about/guidelines.xhtml

For peer review only

12. Carson MG, Mitchell GJ. The experience of living with persistent pain. Journal of advanced nursing. 1998;28(6):1242-8.
13. De Souza L, Oliver Frank A. Patients' experiences of the impact of chronic back pain on family life and work. Disability and Rehabilitation. 2011;33(4):310-8.
14. Gillsjö C, Nässén K, Berglund M. Suffering in silence: a qualitative study of older adults' experiences of living with long-term musculoskeletal pain at home. European journal of ageing. 2021;18(1):55-63.
15. Andersson C, Mårtensson L. Women's experiences of being in the sick leave process. Scandinavian journal of occupational therapy. 2021;28(6):488-97.
16. Severinsen A, Midtgaard J, Backhausen MG, Broberg L, Hegaard HK. Pregnant women's experiences with sick leave caused by low back pain. A qualitative study. Work (Reading, Mass). 2019;64(2):271-81.
17. Lindbäck C, Nordgren L. To be on sick-leave due to heart failure: a qualitative perspective. Disabil Rehabil. 2015;37(19):1732-8.
18. Lännerström L, Wallman T, Holmström IK. Losing independence – the lived experience of being long-term sick-listed. BMC Public Health. 2013;13(1):745.
19. LoMartire R, Björk M, Dahlström Ö, Constan L, Frumento P, Vixner L, et al. The value of interdisciplinary treatment for sickness absence in chronic pain: A nationwide register-based cohort study. 2021;25(10):2190-201.
20. Wainwright E, Wainwright D, Keogh E, Eccleston C. Return to work with chronic pain: employers' and employees' views. Occupational Medicine. 2013;63(7):501-6.
21. Grant M, Rees S, Underwood M, Froud R. Obstacles to returning to work with chronic pain: in-depth interviews with people who are off work due to chronic pain and employers. BMC musculoskeletal disorders. 2019;20(1):486.
22. Nortvedt L, Lohne V, Kumar BN, Hansen HP. A lonely life--A qualitative study of immigrant women on long-term sick leave in Norway. International journal of nursing studies. 2016;54:54-64.
23. Nortvedt L, Hansen HP, Kumar BN, Lohne V. Caught in suffering bodies: a qualitative study of immigrant women on long-term sick leave in Norway. Journal of clinical nursing. 2015;24(21-22):3266-75.
24. Brataas HV, Evensen AE. Life stories of people on sick leave from work because of mild mental illness, pain and fatigue. Work (Reading, Mass). 2015;53(2):285-91.
25. O'Brien BC, Harris IB, Beckman TJ, Reed DA, Cook DA. Standards for Reporting Qualitative Research: A Synthesis of Recommendations. 2014;89(9):1245-51.
26. Ricoeur P. Interpretation Theory: Discourse and the Surplus of Meaning: Texas Christian University Press; 1976.
27. Lindseth A, Norberg A. A phenomenological hermeneutical method for researching lived experience. Scandinavian Journal of Caring Sciences. 2004;18(2):145-53.
28. Alsén S, Ali L, Ekman I, Fors A. Facing a blind alley - Experiences of stress-related exhaustion: a qualitative study. BMJ open. 2020;10(9):e038230.
29. Wallström S, Ulin K, Omerovic E, Ekman I. Symptoms in patients with takotsubo syndrome: a qualitative interview study. BMJ open. 2016;6(10):e011820.
30. Söderberg S, Lundman B, Norberg A. Struggling for dignity: the meaning of women's experiences of living with fibromyalgia. Qualitative health research. 1999;9(5):575-87.
31. Polkinghorne DE. Phenomenological research methods. Existential-phenomenological perspectives in psychology: Exploring the breadth of human experience. New York, NY, US: Plenum Press; 1989. p. 41-60.
32. Association WM. World Medical Association Declaration of Helsinki: Ethical Principles for Medical Research Involving Human Subjects. JAMA. 2013;310(20):2191-4.
33. Ricœur P. Oneself as another. New ed. ed. Blamey K, editor: University of Chicago Press; 1994.

For peer review only

For peer review only - http://bmjopen.bmj.com/site/about/guidelines.xhtml

34. Ekman I. Practising the ethics of person-centred care balancing ethical conviction and moral obligations. Nursing Philosophy. 2022:e12382.
35. van Rhenen W, Schaufeli WB, van Dijk FJ, Blonk RW. Coping and sickness absence. International archives of occupational and environmental health. 2008;81(4):461-72.
36. Svenaeus F. The body uncanny — Further steps towards a phenomenology of illness. Medicine, Health Care and Philosophy. 2000;3(2):125-37.
37. Paulson M, Danielson E, Söderberg S. Struggling for a Tolerable Existence: The Meaning of Men's Lived Experiences of Living with Pain of Fibromyalgia Type. 2002;12(2):238-49.
38. Geneen LJ, Martin DJ, Adams N, Clarke C, Dunbar M, Jones D, et al. Effects of education to facilitate knowledge about chronic pain for adults: a systematic review with meta-analysis. Systematic Reviews. 2015;4(1):132.
39. Lundberg M, Styf J, Bullington J. Experiences of moving with persistent pain--a qualitative study from a patient perspective. Physiotherapy theory and practice. 2007;23(4):199-209.
40. Merleau-Ponty M. Phenomenology of Perception: The Humanities Press; 1962.
41. De Ruddere L, Craig KD. Understanding stigma and chronic pain: a-state-of-the-art review. 2016;157(8):1607-10.
42. Smith BH, Fors EA, Korwisi B, Barke A, Cameron P, Colvin L, et al. The IASP classification of chronic pain for ICD-11: applicability in primary care. Pain (Amsterdam). 2019;160(1):83-7.
43. Wijnhoven HA, de Vet HC, Picavet HS. Prevalence of musculoskeletal disorders is systematically higher in women than in men. The Clinical journal of pain. 2006;22(8):717-24.
44. Sandelowski M. Sample size in qualitative research. Res Nurs Health. 1995;18(2):179-83.
45. Thunberg S, Arnell L. Pioneering the use of technologies in qualitative research – A research review of the use of digital interviews. International Journal of Social Research Methodology. 2021:1-12.

For peer review only

For peer review only - http://bmjopen.bmj.com/site/about/guidelines.xhtml

GÖTEBORGS UNIVERSITET

SAHLGRENSKA ACADEMY

- **Introduction**

Presentation of the researchers, the project and its aim. Information about confidentiality, consent and withdrawal. Opportunity for the participants to ask questions.

- **Opening questions**
  - I understand you have been living in pain for a long time. Would you tell me about what this time has been like for you?
  - In what way has being on sick leave and not being able to work affected your life?
  - What would you say has been the biggest challenge for you as the person you think you are or want to be? Has being on sick leave changed the way you look at yourself?
  - Are there things that could have prevented you from being on sick leave?

- **Work and support**
  - Having pain for a long time often affects the ability to work. In what way would you say that your pain has influenced your work situation?
  - Everyone is different; some people want support to cope with their situation, and others feel they have the support they need to manage. How is it for you? What support did you need to deal with your situation?
  - Do you feel that you have been given the opportunity for such support or adjustment at your workplace? If so, what has that support meant to you?

- **Self-efficacy and confidence in one's ability**
  - It is common to lose confidence in your abilities when you have been on sick leave for a long time. What do you think about that? Do you feel you can influence your situation yourself?
  - Being on sick leave and living with pain can also affect how you feel about your body. Do you feel you can trust your body to do what you want to do? What are your thoughts about that?
  - What do you think about the situation you are in today? How does your pain affect the goals and desires that you have in life?

- **Activity and participation**
  - Living with pain for a long time can often affect different aspects of everyday life. How do you feel it affects your leisure activities or social contacts?
  - How has your pain affected your ability to sleep, rest or recover?

- **Conclusion**
  - Do you have any other thoughts or reflections about your situation that we have not discussed today and that you would like to add?

For peer review only

Reporting checklist for qualitative study.

Based on the SRQR guidelines.

Instructions to authors

Complete this checklist by entering the page numbers from your manuscript where readers will find each of the items listed below.

Your article may not currently address all the items on the checklist. Please modify your text to include the missing information. If you are certain that an item does not apply, please write "n/a" and provide a short explanation.

Upload your completed checklist as an extra file when you submit to a journal.

In your methods section, say that you used the SRQRreporting guidelines, and cite them as:

O'Brien BC, Harris IB, Beckman TJ, Reed DA, Cook DA. Standards for reporting qualitative research: a synthesis of recommendations. Acad Med. 2014;89(9):1245-1251.

		Reporting Item	Page Number
**Title**			
	#1	Concise description of the nature and topic of the study identifying the study as qualitative or indicating the approach (e.g. ethnography, grounded theory) or data collection methods (e.g. interview, focus group) is recommended	1
**Abstract**			
	#2	Summary of the key elements of the study using the abstract format of the intended publication; typically includes background, purpose, methods, results and conclusions	2
**Introduction**			
Problem formulation	#3	Description and signifcance of the problem / phenomenon studied: review of relevant theory and empirical work; problem statement	3-5

For peer review only

Purpose or research question	#4	Purpose of the study and specific objectives or questions	5
**Methods**			
Qualitative approach and research paradigm	#5	Qualitative approach (e.g. ethnography, grounded theory, case study, phenomenolgy, narrative research) and guiding theory if appropriate; identifying the research paradigm (e.g. postpositivist, constructivist / interpretivist) is also recommended; rationale. The rationale should briefly discuss the justification for choosing that theory, approach, method or technique rather than other options available; the assumptions and limitations implicit in those choices and how those choices influence study conclusions and transferability. As appropriate the rationale for several items might be discussed together.	5
Researcher characteristics and reflexivity	#6	Researchers' characteristics that may influence the research, including personal attributes, qualifications / experience, relationship with participants, assumptions and / or presuppositions; potential or actual interaction between researchers' characteristics and the research questions, approach, methods, results and / or transferability	7
Context	#7	Setting / site and salient contextual factors; rationale	5
Sampling strategy	#8	How and why research participants, documents, or events were selected; criteria for deciding when no further sampling was necessary (e.g. sampling saturation); rationale	5-6, 19
Ethical issues pertaining to human subjects	#9	Documentation of approval by an appropriate ethics review board and participant consent, or explanation for lack thereof; other confidentiality and data security issues	7
Data collection methods	#10	Types of data collected; details of data collection procedures including (as appropriate) start and stop dates of data collection and analysis, iterative process, triangulation of sources / methods, and modification of procedures in response to evolving study findings; rationale	6

For peer review only

Data collection instruments and technologies	#11	Description of instruments (e.g. interview guides, questionnaires) and devices (e.g. audio recorders) used for data collection; if / how the instruments(s) changed over the course of the study	6
Units of study	#12	Number and relevant characteristics of participants, documents, or events included in the study; level of participation (could be reported in results)	7-8
Data processing	#13	Methods for processing data prior to and during analysis, including transcription, data entry, data management and security, verification of data integrity, data coding, and anonymisation / deidentification of excerpts	6-7
Data analysis	#14	Process by which inferences, themes, etc. were identified and developed, including the researchers involved in data analysis; usually references a specific paradigm or approach; rationale	6-7
Techniques to enhance trustworthiness	#15	Techniques to enhance trustworthiness and credibility of data analysis (e.g. member checking, audit trail, triangulation); rationale	6-7
**Results/findings**			
Syntheses and interpretation	#16	Main findings (e.g. interpretations, inferences, and themes); might include development of a theory or model, or integration with prior research or theory	7-15
Links to empirical data	#17	Evidence (e.g. quotes, field notes, text excerpts, photographs) to substantiate analytic findings	7-14
**Discussion**			
Intergration with prior work, implications, transferability and contribution(s) to the field	#18	Short summary of main findings; explanation of how findings and conclusions connect to, support, elaborate on, or challenge conclusions of earlier scholarship; discussion of scope of application / generalizability; identification of unique contributions(s) to scholarship in a discipline or field	15-20
Limitations	#19	Trustworthiness and limitations of findings	19-20

For peer review only

Other

Conflicts of interest	#20	Potential sources of influence of perceived influence on study conduct and conclusions; how these were managed	21
Funding	#21	Sources of funding and other support; role of funders in data collection, interpretation and reporting	21

The SRQR checklist is distributed with permission of Wolters Kluwer © 2014 by the Association of American Medical Colleges. This checklist was completed on 14. June 2019 using https://www.goodreports.org/, a tool made by the EQUATOR Network in collaboration with Penelope.ai

For peer review only
